# Lipid parameters, adipose tissue distribution and prognosis prediction in chronic kidney Disease patients

**DOI:** 10.1186/s12944-024-02004-4

**Published:** 2024-01-08

**Authors:** Hui-fen Chen, Bing-jie Xiao, Lin-yi Chen, Wen-wei OuYang, Xian-long Zhang, Zhi-ren He, Li-zhe Fu, Fang Tang, Xiao-na Tang, Xu-sheng Liu, Yi-fan Wu

**Affiliations:** 1 The Second Clinical College, Guangzhou University of Chinese Medicine, Guangzhou, Guangdong China; 2 Key Unit of Methodology in Clinical Research, The Second Affiliated Hospital of Guangzhou University of Chinese Medicine (Guangdong Provincial Hospital of Chinese Medicine), Guangzhou, China; 3 Global Health - Health Systems and Policy, Department of Global Public Health, Karolinska Institute, Stockholm, Sweden; 4 Renal Division, The Second Affiliated Hospital of Guangzhou University of Chinese Medicine (Guangdong Provincial Hospital of Chinese Medicine), Guangzhou, Guangdong China; 5 Chronic Disease Management Outpatient Clinic, The Second Affiliated Hospital of Guangzhou, University of Chinese Medicine (Guangdong Provincial Hospital of Chinese Medicine), Guangzhou, China; 6 Bao’an Traditional Chinese Medicine Hospital, Guangzhou University of Chinese Medicine, Shenzhen, China

**Keywords:** Chronic kidney disease, Lipid profiles, Lipid distribution

## Abstract

**Background:**

Lipid management in clinic is critical to the prevention and treatment of Chronic kidney disease (CKD), while the manifestations of lipid indicators vary in types and have flexible association with CKD prognosis.

**Purpose:**

Explore the associations between the widely used indicators of lipid metabolism and their distribution in clinic and CKD prognosis; provide a reference for lipid management and inform treatment decisions for patients with non-dialysis CKD stage 3–5.

**Methods:**

This is a retrospective cohort study utilizing the Self-Management Program for Patients with Chronic Kidney Disease Cohort (SMP-CKD) database of 794 individuals with CKD stages 3–5. It covers demographic data, clinical diagnosis and medical history collection, laboratory results, circulating lipid profiles and lipid distribution assessments. Primary endpoint was defined as a composite outcome(the initiation of chronic dialysis or renal transplantation, sustained decline of 40% or more in estimated glomerular filtration rate (eGFR), doubled of serum creatinine (SCr) from the baseline, eGFR less than 5 mL/min/1.73m^2^, or all-cause mortality). Exposure variables were circulating lipid profiles and lipid distribution measurements. Association were assessed using Relative risks (RRs) (95% confidence intervals (CIs)) computed by multivariate Poisson models combined with least absolute shrinkage and selection operator (LASSO) regression according to categories of lipid manifestations. The best model was selected via akaike information criterion (AIC), area under curve (AUC), receiver operating characteristic curve (ROC) and net reclassification index (NRI). Subgroup analysis and sensitivity analysis were performed to assess the interaction effects and robustness..

**Results:**

255 individuals reached the composite outcome. Median follow-up duration was 2.03 [1.06, 3.19] years. Median age was 58.8 [48.7, 67.2] years with a median eGFR of 33.7 [17.6, 47.8] ml/min/1.73 m^2^. Five dataset were built after multiple imputation and five category-based Possion models were constructed for each dataset. Model 5 across five datasets had the best fitness with smallest AIC and largest AUC. The pooled results of Model 5 showed that total cholesterol (TC) (RR (95%CI) (per mmol/L) :1.143[1.023,1.278], *P* = 0.018) and percentage of body fat (PBF) (RR (95%CI) (per percentage):0.976[0.961,0.992], *P* = 0.003) were significant factors of composite outcome. The results indicated that comprehensive consideration of lipid metabolism and fat distribution is more critical in the prediction of CKD prognosis..

**Conclusion:**

Comprehensive consideration of lipid manifestations is optimal in predicting the prognosis of individuals with non-dialysis CKD stages 3–5.

**Supplementary Information:**

The online version contains supplementary material available at 10.1186/s12944-024-02004-4.

## Introduction

Chronic kidney disease (CKD) affects roughly 7.0–34.3% of adults globally [1]. It resulted in 1.2 million deaths in 2017 and the Global Burden of Disease Study estimates that this figure will reach 2.2 million by 2040 [2]. Once the condition progresses to end-stage renal disease (ESRD), renal replacement therapy (RRT) is needed to prolong life, but this entails lower health-related quality of life and major financial costs [3]. Therefore, thwarting the initiation of RRT is a critical goal in advanced CKD treatment.

Clinical complexity is associated with higher risk of dialysis and lower survival rate among people with CKD stages 3–5 [4]. The prevention and treatment of various complications is significant means of delaying CKD progression, and lipid metabolism management is one important means of accomplishing this. People with CKD are prone to dyslipidemia. On the one hand, persistent proteinuria stimulates compensatory hepatic synthesis of excessive lipoprotein [5]; on the another hand, the damaged kidney function leads to decreased clearance rates and abnormal cholesterol transport, which can precipitate or aggravate kidney dysfunction and cell damage in turn [5]. Lipid disorder is also relevant to other etiologies in CKD, such as cardiovascular disease (CVD), diabetes and hypertension [6–9]. The vicious lipid-mediated renal injury cycle obviously worsens overall prognosis for individuals with CKD.

Therefore, research is needed to identify lipid abnormalities, explore their effects on CKD prognosis and provide targeted treatments for condition management. However, lipid metabolism and fat distribution disorders often manifest as abnormalities in plasma (e.g., total cholesterol (TC), triglycerides (TG), lipoprotein), machine (e.g., whole, local fat) and anthropometric measures (e.g., body mass index (BMI), mid-arm circumference (MUAC), triceps skinfold thickness (TSKF)) in clinic. Additionally, previous studies have shown a flexible correlation between these manifestations and CKD prognosis. According to Lee C. et al. higher LDL-C levels are associated with 1.28 to 2.21-fold higher risk of adverse kidney outcomes, compared to a group with LDL-C less than 70 mg/dl. Other studies have shown that LDL-C is not an independent predictor of CKD prognosis [10, 11]. A similar discrepancy has been found with anthropometric measurements. Among individuals with CKD stages 3–5, percentage of body fat(PBF) was not a predictive factor for ESRD, even though there was a U-shaped trend [12]. BMI had a positive correlation with renal outcomes in a group with 15 < BMI < 20 kg/m^2^ and a group with 20 < BMI < 22.5 kg/m^2^ in CKD stages 4–5 [13]. However, Kataoka H. et al. only obtained a similar result in a group with BMI ≥ 25 kg/m^2^ [14].

We believe that divergent clinical significance in lipid-related parameters led to various effects on CKD prognosis. Many studies have explored the effects of a single clinical manifestation, or a category of them, on CKD prognosis, and ultimately the results have been inconclusive. Inconsistent conclusions have also been due to differences across populations. Comprehensively considering these parameters might lead to more accurate prediction of CKD prognosis, and provide a references for early therapies. In this study, we utilized the Self-Management Program for Patients with Chronic Kidney Disease Cohort (SMP-CKD cohort) database to explore the associations between various classifications of lipid parameters and CKD prognosis.

## Methods

### Study design

The SMP-CKD cohort is an ongoing, multi-center observational cohort under study at the Guangdong Provincial Hospital of Chinese Medicine (GPHCM) (Ethics approval No. 2019-153-01; Chinese Clinical Trial Registry No. ChiCTR1900024633). It has an expected duration of approximately 10 years.

The SMP-CKD study is an ambispective cohort study with a retrospective period from July 2015 to July 2019 with a prospective period from August 2019 to August 2024. Its aim is to observe the effects of patients’ self-management ability on CKD prognosis. Chinese patients with CKD stage 1–5, aged 18–80 years, with written signed informed consent are eligible for SMP-CKD. Exclusion criteria include participants with mental health problems, those who are unable to cooperate with clinical staff, those with a history of dialysis or renal replacement and those with severe fluid retention disorders. CKD is defined as a glomerular filtration rate (GFR) below 60mL/min/1.73m^2^ and/or having biomarkers signifying renal injury for at least 3 months. CKD stages are calculated according to the CKD Epidemiology Collaboration (CKD-EPI) creatinine equation [15]. As of December 31, 2022, the cohort had included 3,312 people with CKD stages 1–5. Details on the SMP-CKD have been previously described [16].

For the present study, we retrospectively collected information on individuals with CKD stages 3–5 who had been enrolled between July 1, 2015 and December 31, 2022 at an SMP-CKD center. Participants with incomplete lipid-related parameters at the baseline, with severe fluid retention at the baseline, and those with survival times less than 3 months, were excluded. Composite outcome was defined as prognosis for patients with CKD, including time to first ESRD onset event (defined as the initiation of chronic dialysis or renal transplantation), sustained decline of 40% or more in eGFR, doubled of SCr from the baseline, eGFR less than 5 mL/min/1.73m^2^, or all-cause mortality. Patients were followed to the end of the follow-up time period (December 31, 2022) or loss to follow-up(defined as unobservable during the time-at-risk period).

### Definition of exposures

Our exposures were circulating lipid profiles and fat distribution measurements. The lipid fractions were comprised of TG, TC, high-density lipoprotein cholesterol (HDL-C), and low-density lipoprotein cholesterol (LDL-C). Adipose tissue distribution was ascertained through manual measurement and multi-frequency bioimpedance assessment (InBody Europe BV. www.inbody.de. Accessed 29 Oct 2021). Overall fat distribution was evaluated by BMI, body fat mass (BFM), fat mass index (FMI) and PBF. Localized fat distributions were estimated by TSKF, MUAC and visceral fat area (VFA). The MUAC was measured with a non-extensible, flexible tape, and TSKF was measured at the same arm with a skinfold caliper (to the nearest 0.1 centimeters). Fat free mass (FFM), FFM index (FFMI), total body water (TBW), intracellular water (ICW), and extracellular water (ECW) were also included as covariates to minimize the influence of confounders.

### Ascertaining covariates

Baseline information was collected from a pre-designed questionnaire and clinical records. Demographic data included age (continuous), sex (male vs. female), self-reported measures (marital status, alcohol use, smoking status, education levels, self-care capacity), clinical diagnosis (protopathy and comorbidities) and medication in use (angiotensin-converting enzyme inhibitors (ACEI) or angiotensin receptor blockers (ARB), other antihypertensives drugs, uric acid (UA) lowering drugs, lipid-lowering drugs, folic acid supplements, iron polysaccharide complex or erythropoietin (EPO)).

Protopathy was classified as primary glomerulonephritides (including chronic nephritis, nephropathy syndrome and IgA nephropathy), hypertensive renal disease, diabeticnephropathy, other secondary nephrosis (e.g., systemic lupus erythematosus nephritis, Henoch-Schonlein purpura, Hepatitis B virus-associated nephritis and obstructive nephropathy), or unidentified etiology.

Management of hypertension, diabetes and hyperuricaemia were classified as “had specific comorbidity” (with abnormal or normal level, or without regular observation) and “without specific comorbidity” by collecting self-reported comorbidities, baseline blood pressure (BP), fasting glucose (Glu), uric acid (UA) levels and medication in use. History of CVDs was identified through cardiac ultrasound and electrocardiogram. Laboratory covariates included hemoglobin (Hb), UA, glycated hemoglobin (HbA1c), Glu, total carbon dioxide (TCO_2_), urea, K^+^, Na^+^, Ca^2+^, P, albumin (ALB), eGFR and urine protein-to-creatinine ratio (UPCR).

### Statistical analyses

For missing values and outliers, the whole dataset was split into complete cases and incomplete cases after encoding all variables. In complete cases, outliers of continuous variables were winsorized with 0.1 cut-offs at each tail. Then, missing baseline covariates were imputed with multiple imputation for the whole dataset (with the mice R package) and pooled the estimators across imputed datasets. Glu and HbA1c with missing rates exceeding 20% were excluded. Percentage of missing data was shown in Supplement file 1.

For baseline characteristic description, continuous variables were described using mean ± standard deviation (SD) for normal distributions and median (interquartile ranges) for non-normal distributions. Categorical variables were reported by proportions. Risk of composite outcomes across variables was estimated using Poisson regression analysis (with the biostat3 R package) because Schoenfeld residuals (with the survminer R package) showed violation of the proportional hazard assumption in some observed variables (Supplement file 2). Martingale residuals was applied to detect non-linearity in relationship between the log hazard and the covariates. First, to develop time split data, an offset for the log follow-up duration was developed by splitting the time into several timescales with the survSplit function. The survival rate for each timescale was calculated via the survRate function. Then, univariate Poisson models in each dataset were constructed with the offset and timescales (as factors). Independent variables with *P* < 0.10 were included in multivariable-adjusted Poisson models. Least absolute shrinkage and selection operator (LASSO) regression combined with cross-validation was used to select features. Association were assessed using Relative risks(RRs) (95% confidence intervals (CIs)). The models’ accuracy and benefits were compared by Akaike information criterion (AIC), *P* for the receiver operating curve (ROC) and the area under the receiver operating curve (AUC). Net reclassification index (NRI) was used to assess added predictive utility based on prespecified risk thresholds of 20% and 40%, and 95%CIs were estimated by bootstrapping [17, 18].

For subgroup analysis, multivariable-adjusted Poisson models with strata of sex, age, CKD stages, with or without hypertension, with or without diabetes and use of lipid-lowering drugs were also constructed. Forest plots were used to show the relative risk (RRs) with 95% (CIs) and interaction with subgroups. For sensitivity analysis, a Poisson model was constructed with complete cases to assess the robustness of our results.

**Fig. 1:**
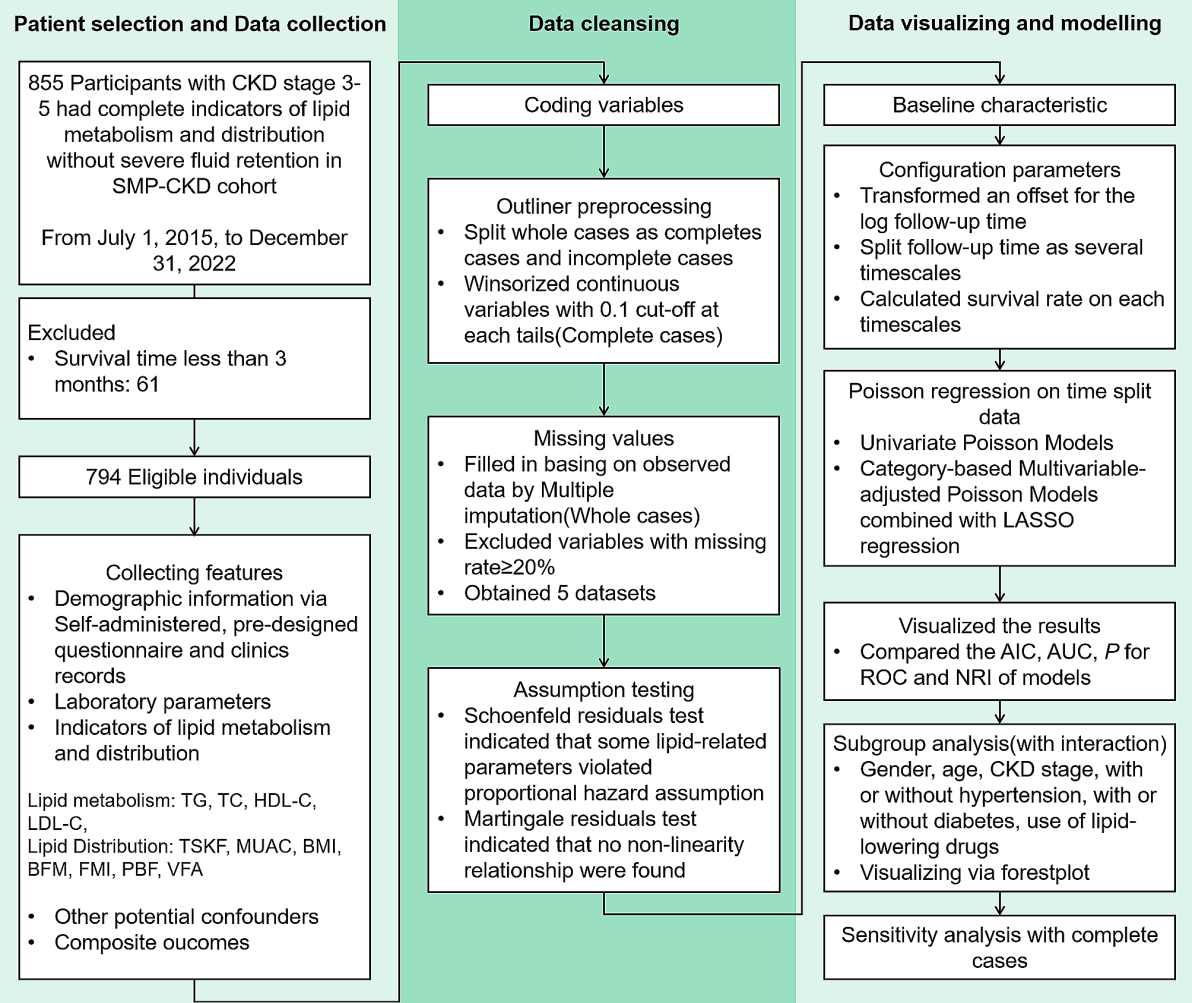
Details on patients selection and statistical analyses

All statistical tests were two-sided, and ***P*** values < 0.05 were considered statistically significant. Statistical analysis was performed using R version 4.2.1. Details on patients selection and statistical analyses are illustrated in Fig. 1.

## Results

### Baseline characteristics

855 individuals with CKD stage 3–5 with complete indicators of lipid metabolism and distribution were registered in a single-center SMP-CKD cohort between July 1, 2015 and December 31, 2022. 61 survived less than 3 months were excluded. A total of 794 individuals (365 (45.97%) were females; median age of 58.8 [48.7, 67.2] years with moderate to severe CKD (median eGFR of 33.7 [17.6, 47.8] ml/min/1.73 m^2^, median urea of 10.87 [7.92, 15.75] mmol/L and median UPCR of 0.87 [0.27, 2.03] mg/g) were included in the analysis. 270 (34.01%) were diagnosed with primary glomerulonephritides. 44 (5.54%) had hypertensive renal disease and 110 (13.85%) had diabetic nephropathy. 118 (14.86%) had other secondary nephrosis, and 252 (31.74%) were estimated to have unknown protopathy.

655 (82.49%) of patients had a history of hypertension, 585 (73.68%) had a history of hyperuricemia, and 334 (42.06%) were diagnosed with diabetic. 161 (20.28%) had a history of CVDs. 336 (42.32%) of them were reported the use of ACEI/ARB and 272 (34.36%) were using lipid-lowering therapy. Baseline characteristics of the study population are described in Table 1.

**Table 1:** Baseline cohort characteristics

Variables		N(%)/Mean(SD)/Median[25th,75th]	Variables	N(%)/Mean(SD)/Median[25th,75th]
Age		58.8 [48.7, 67.2]	Medication	
Gender	Male	429(54.03)	ACEI/ARB	336(42.32)
	Female	365(45.97)	Other antihypertensive drugs	432(54.41)
Marital status	Unmarried	40(5.04)	Hypoglycemic agents	171(21.54)
	Married	737(94.85)	Urate-lowering drugs	404(50.89)
Alcohol Consumption	No	779(98.11)	Lipid-lowering drugs	272(34.36)
	Yes	15(1.89)	Folic acid tablets	86(10.83)
Current Smoker	No	725(91.31)	Polysaccharide iron	122(15.37)
	Yes	69(8.69)	EPO	133(16.75)
Education	Elementary school	118(14.90)	Hb, g/L	119.64(21.75)
	Junior high school	229(28.91)	TCO_2_, mmol/L	22.90 [20.78, 25.00]
	High school	257(32.45)	UA, µmmol/L	427.00 [370.75, 501.75]
	College degree or above	188(23.74)	Urea, mmol/L	10.87 [7.92, 15.75]
Without self-care capacity	Yes	52(6.55)	ALB, g/L	43.60 [40.28, 46.10]
	No	742(93.45)	eGFR, ml/min/1.73 m^2^	33.7 [17.6, 47.8]
Protopathy	Primary Glomerulonephritides	270(34.01)	K^+^, mmol/L	4.48 [4.18, 4.83]
	Hypertensive Renal Disease	44(5.54)	Na^+^, mmol/L	141.00 [139.00, 142.00]
	Diabetic nephropathy	110(13.85)	Ca^2+^, mmol/L	2.33 [2.25, 2.42]
	Others	118(14.86)	P, mmol/L	1.28 [1.12, 1.44]
	Unknown	252(31.74)	UPCR, mg/g	0.87[0.27,2.03]
Comorbidity			TG, mmol/L	1.49 [1.10, 2.18]
Hypertension management			TC, mmol/L	4.79 [4.00, 5.57]
Hypertension with normal BP		164(20.66)	HDL-C, mmol/L	1.26 [1.00, 1.52]
Hypertension with abnormal BP		396(49.87)	LDL-C, mmol/L	3.03 [2.30, 3.73]
Hypertension with without regular assessment		95(11.97)	TSKF, cm	1.40 [1.00, 1.90]
Non-hypertension		139(17.51)	MUAC, cm	27.00 [25.40, 29.00]
Glu management			BMI, kg/m^2^	22.80 [20.60, 25.03]
Diabetes with normal Glu		142(17.89)	BFM, kg	15.45 [11.35, 19.90]
Diabetes with abnormal Glu		117(14.74)	FMI, %	5.90 [4.30, 7.50]
Diabetes without regular assessment		75(9.45)	PBF, %	26.19(8.38)
Non-diabetes		460(57.94)	VFA, cm^2^	68.70 [51.87, 91.63]
UA management			TBW, kg/L	32.65 [27.80, 37.51]
Hyperuricemia with normal uric acid		198(24.94)	ICW, kg/L	20.00 [16.90, 22.90]
Hyperuricemia with anormal uric acid		387(48.74)	ECW, kg/L	12.65 [10.90, 14.60]
Non-hyperuricemia		206(25.95)	FFM, kg	44.20 [37.90, 51.00]
With history of cardiovascular disease		161(20.28)	FFMI, %	16.83(2.05)

Note:Primary Glomerulonephritides included chronic nephritis, nephropathy syndrome and IgA nephropathy.Other secondary nephrosis included systemic lupus erythematosus nephritis, Henoch-Schonlein purpura,Hepatitis B virus-associated nephritis and obstructive nephropathy, etc. Blood Pressure, BP; Angiotensin converting enzyme, ACE; Angiotensin receptor blocker, ARB; Uric acid, UA; Erythropoietin, EPO; Hemoglobin, Hb; Triglyceride, TG; total cholesterol, TC; high-density lipoprotein cholesterol,HDL-C; low-density lipoprotein cholesterol, LDL-C; Total carbon dioxide, TCO2; albumin, ALB; Urine protein-to-creatinine, UPCR; Body Mass Index, BMI; triceps skinfold thickness, TSKF; mid-arm circumference, MUAC; Body Fat Mass, BFM; Fat Mass Index, FMI; Percent Body Fat, PBF; Visceral Fat Area, VFA; Total Body Water, TBW; Intracellular Water, ICW; Extracellular Water, ECW; Fat Free Mass, FFM; Fat Free Mass Index, FFMI.

### Poisson regression on time-split data

During a median follow-up of 2.03 [1.06, 3.19] years, 255 subjects reached the composite outcome. We split the follow-up duration into seven timescales. Table 2 described the survival rate of each timescale’s composite endpoint. Taking the censored samples as reference, 78 (42.86%) and 92 (42.79%) of events occurred with crude survival rates of 0.108 [0.086, 0.135] and 0.186 [0.150, 0.228], respectively, in the first and second timescale. The events occurred in the 3 to 6 timescale were 44 (25.29%) (0.144 [0.104, 0.193]), 32 (29.09%) (0.197 [0.135, 0.278]), 7 (11.67%) (0.088 [0.035, 0.181]) and 2 (3.92%) (0.069 [0.008, 0.249]), respectively. No events occurred in the last timescale.

**Table 2:** Survial rate of composite endpoint in timescales

Follow-up Timescales	Person-years	Events n(%)	Crude Survival Rate[95%CI]
Timeband = 1	182	78(42.86)	0.108[0.086,0.135]
Timeband = 2	215	92(42.79)	0.186[0.150,0.228]
Timeband = 3	174	44(25.29)	0.144[0.104,0.193]
Timeband = 4	110	32(29.09)	0.197[0.135,0.278]
Timeband = 5	60	7(11.67)	0.088[0.035,0.181]
Timeband = 6	51	2(3.92)	0.069[0.008,0.249]
Timeband = 7	2	0(0)	0.000[0.000,0.835]

After adjusting the offset and timescales (as factors), the univariate Poisson models in five datasets generated variables with *P* < 0.10 including covariates (age, marital status, Hb, TCO_2_, Urea, ALB, UPCR, eGFR, K^+^, Na^+^, Ca^2+^, P and ECW), circulating lipid profile (TC), manual measurements (TSKF, MUAC and BMI) and body composition (BFM, PBF, VFA and FMI). The results of univariate Poisson regression for each dataset are described in Supplement file 3.

The category-based multivariable-adjusted Poisson models combined with LASSO regression were constructed to compare the associations of various lipid parameters with prognosis. Taking Model 1 (containing only covariates) as a basis, Model 2 added lipid fractions, Model 3 added manual measurements and Model 4 added body composition parameters. Model 5 contained all variables with *P* < 0.10. After LASSO regression, Model 5 across five datasets had the best fitness with smallest AIC and largest AUC. Hence, we pooled the results of Model 5 in Table 3. Results of Dataset 1–5 were detailed in Supplement file 4 and 5. The pooled results of Model 5 across 5 datasets showed that TC and PBF were significantly independent factors associated with composite outcome (TC:RR (95%CI) (per mmol/L) :1.143[1.023,1.278], *P* = 0.018). PBF: RR (95%CI) (per percentage):0.976[0.961,0.992], *P* = 0.003).

**Table 3:** Pooled results of Model 5

	Pooled	
Variables	RRs[95%CI]	*P*
TC, mmol/L	1.143[1.023,1.278]	0.018
BMI, kg/m^2^	0.984[0.948,1.020]	0.412
PBF, %	0.976[0.961,0.992]	0.003
Marriage	1.200[0.742,1.940]	0.457
Age, year	0.989[0.980,0.999]	0.024
Hb, g/L	0.991[0.983,0.999]	0.034
TCO2, mmol/L	0.986[0.946,1.027]	0.496
Urea, mmol/L	1.014[0.985,1.044]	0.347
UPCR, mg/g	1.163[1.082,1.250]	0.000
eGFR, ml/min/1.73 m^2^	0.956[0.943,0.969]	0.000
K^+^, mmol/L	1.015[0.926,1.514]	0.178
Na^+^, mmol/L	0.958[0.910,1.009]	0.107
Ca^2+^, mmol/L	0.836[0.353,1.980]	0.685
P, mmol/L	2.545[1.468,4.414]	0.001

### Subgroup analysis

**Fig. 2:**
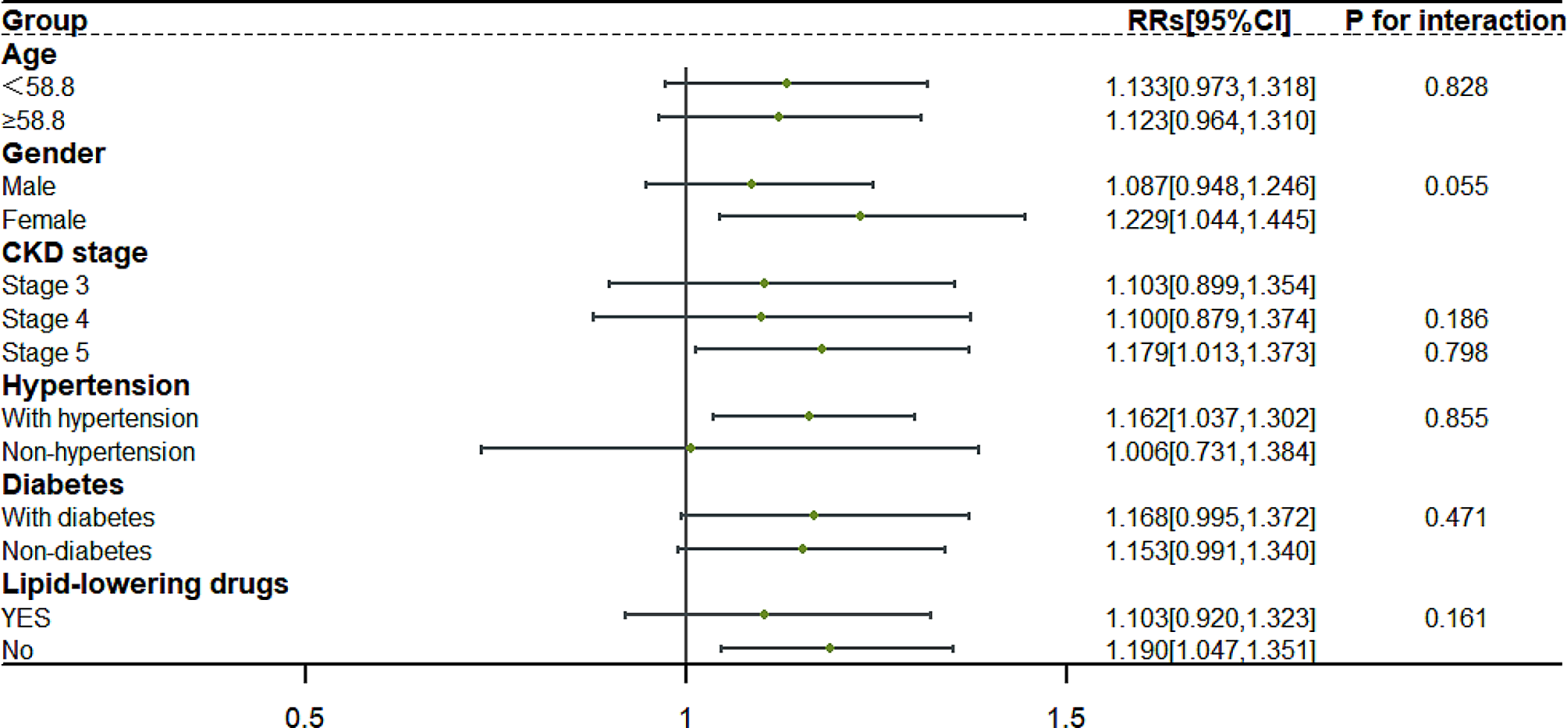
Subgroup analyses of TC

Subgroup analysis with strata of sex, age (group by median), CKD stages, with or without hypertension, with or without diabetes and use of lipid lowering drugs, were constructed on the basis of Model 5 and was pooled across five datasets (Supplement file 6). In Fig. 2, TC was a relative risk factor for the group with females (RR (95%CI) (per mmol/L) :1.229[1.214,1.243], *P* = 0.013), the group with CKD stage 5 (RR (95%CI) (per mmol/L) :1.179[1.166,1.193], *P* = 0.034), the group with hypertension (RR (95%CI) (per mmol/L) :1.162[1.155,1.169], *P* = 0.010), and those who were not using lipid lowering drugs (RR (95% CI) (per mmol/L) : 1.190[1.181,1.199], *P* = 0.008).

**Fig. 3:**
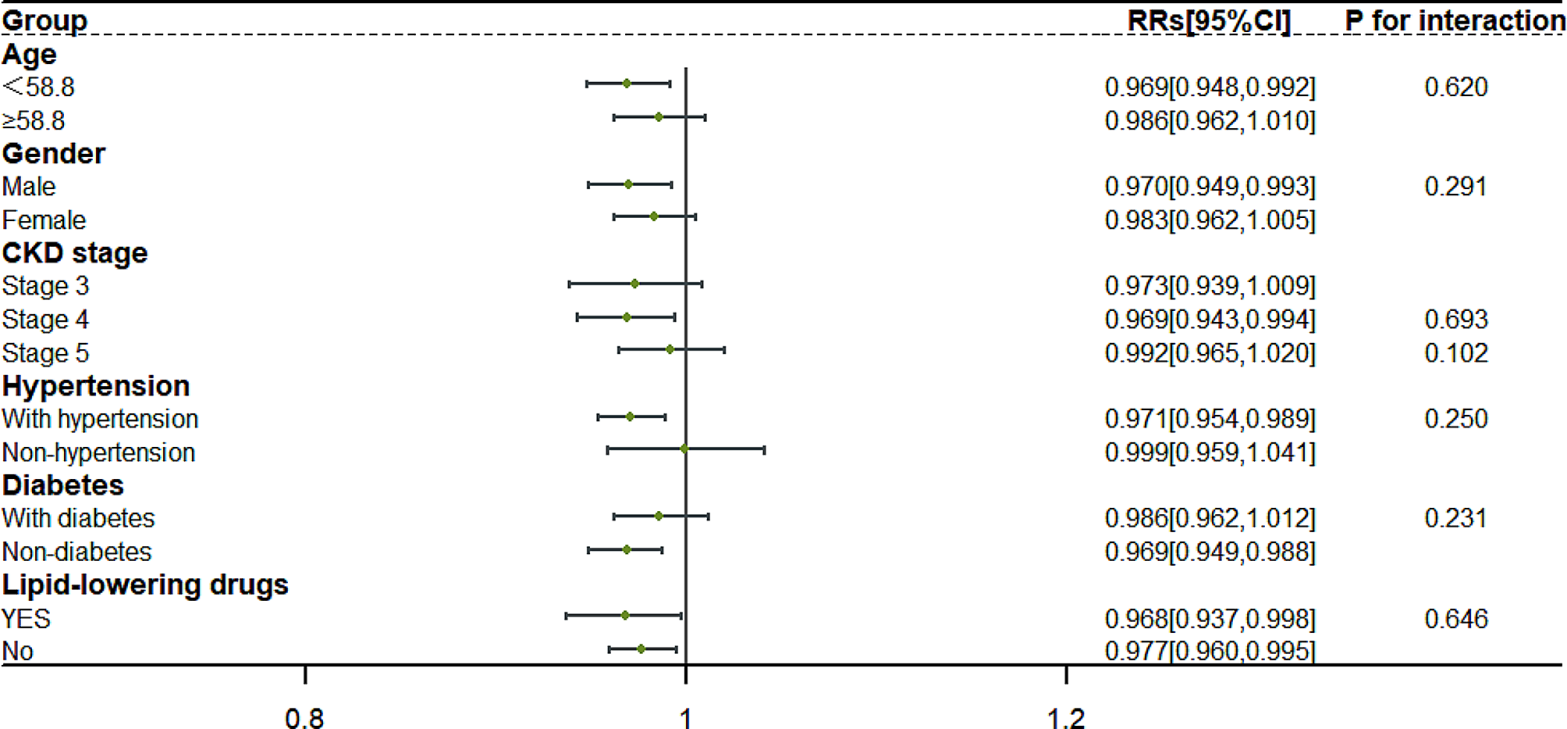
Subgroup analyses of PBF

In Fig. 3, PBF was a relative protective factor of the group with age less than 58.8 years (RR (95%CI) (per percentage) :0.969[0.948,0.992], *P* = 0.007), males (RR(95%CI) (per percentage) :0.970[0.949,0.993], *P* = 0.042), with CKD stage 4 group (RR (95%CI) (per percentage) :0.969[0.943,0.994], *P* = 0.015), the hypertension group (RR (95%CI) (per percentage) :0.971[0.954,0.989], *P* = 0.001), the no diabetes group (RR (95%CI) (per percentage) :0.969[0.949,0.988], *P* = 0.001) and both using or not using lipid lowering drugs (Yes: RR (95% CI) (per percentage) : 0.968[0.937,0.998], *P* = 0.037; No:RR (95% CI) (per percentage) : 0.977[0.96,0.995], *P* = 0.014). No interactions were found in subgroup analysis.

### Sensitivity analysis

For sensitivity analysis, incomplete cases were dropped before repeating the Poisson regression. In the univariate Poisson regression, variables with *P* < 0.10 were covariates (age, Hb, TCO_2_, Urea, ALB, UPCR, eGFR, K^+^, Ca^2+^, P, ECW), manual measurement (TSKF) and body composition (BFM, PBF, VFA, FMI, FFMI). Category-based multivariable-adjusted Poisson models showed that the results of Model 1 were the same as those of Model 2, as were Models 3 and 4. Hence, we displayed the details of Models 1 and 3 in Supplement file 7. Although PBF was still a component of Model 3 after LASSO, both of TC and PBF was not significant factors of composite outcome.

## Discussion

Through a timescale-based Poisson regression analysis on the SMP-CKD cohort, we found that a strong association between composite outcome and both TC (RR (95%CI) (per mmol/L) :1.143 [1.023, 1.278], *P* = 0.018) and PBF (RR (95%CI) (per percentage) :0.976 [0.961, 0.992], *P* = 0.003). The results of sensitivity analysis showed supported more robust effect of PBF than TC on composite outcome. Our study also showed that adding lipid-related parameters to a model that included age, Hb, eGFR, UPCR and other risk factors resulted in more desirable accuracy and slightly better NRI of 0.8%.

Managing LDL-C levels has been the point of interest for thwarting CKD progression in several studies and in guidelines, and there have been recent discoveries in the association between HDL-C, TG and CKD prognosis [19–23]. However, our study showed no significance of LDL-C, HDL-C or TG in univariate Poisson regression. Although TC had stable association with composite outcomes after fully adjusting the other variables, it showed instability in sensitivity analysis. In fact, we further constructed a ratio of LDL-C to HDL-C to explore whether the proportion of LDL-C influenced CKD prognosis, but it still drew an insignificant association in univariate Poisson regression. This revealed that lipid profiles in Chinese patients with CKD might not be as important as those in patients of European decent in thwarting the occurrence of composite outcomes. Observation studies with longer durations and larger sample sizes should be conducted to reveal specific roles of numerous lipid indicators in CKD prognosis.

In recent years, evidence has shown that fat tissue accumulation may be beneficial to survival rate, which has become known as the “obesity paradox” [24]. Numerous studies have revealed that fat storage is a protective factor associated with lower mortality risk in patients with advanced CKD [25, 26]. BMI and PBF are conventional manifestations of fat storage, according to the WHO and Lin TY et al. [25, 27]. In our cohort, PBF had a protective effect on prognosis among people with CKD stages 3–5 and no significant results were found in addition to TC. These results corroborate research which supports obesity paradox [28]. Furthermore, PBF showed more robust effects than TC. Mechanisms of the paradox between fat storage and CKD progression remain unexplained. One potential reason could be that that storing adequate fat tissue provides the potential to prevent malnutrition caused by protein-energy-waste. Similar speculation has been presented in other research. Among dialysis-dependent CKD, fat storage may alleviate weight loss’s negative effects on mortality. Another reason could be that adipose tissue regulates the endocrine system and the processing of uremic toxins [29, 30]. These results may also be related to the range of body shape in the population. In our study, the median BMI was 22.80 [20.60, 25.03]kg/m^2^ and the average PBF was 26.19 ± 8.38%, which does not meet Asian-specific obesity cutoffs [27, 31]. Higher PBF represents a superior nutritional status and leads to better prognosis. However, for the obese, higher PBF levels may bring a different prognosis. Due to our limited sample size, we observed small effects of TC and PBF, and we also did not identify a U-shaped pattern between exposures and the composite outcome.

Considering the effects of multiple lipid-related variables on CKD progression with various time-scales, both lipids in circulation and fat distribution are crucial for patients with non-dialysis-dependent advanced CKD. This is consistent with our initial assumption. Although adding lipid-related parameters to traditional risk factors led to a small NRI of 0.8% at most, the lipid indicators had certain advantages in predicting CKD prognosis. In addition to lipids in plasma, adipose composition warrants more attention from staff in nephrological clinics. The synergistic effects of regulating both of these factors will improve CKD prognosis.

In summary, various lipids measures have different effects on CKD prognosis. A single type of lipids indicators in clinic is not accurate enough. In order to make the most of lipid metabolism and fat distribution on CKD prognosis, and to do so with accuracy and provide effective treatment, we recommend more comprehensive consideration of lipid metabolism and distribution such as manual measures and machine measures receive more comprehensive consideration.

## Conclusion

Composite lipid metabolism and fat distribution have superior accuracy and predictive utility for CKD prognosis among patients with CKD stage 3–5.

## Limitations

Firstly, the aim of this study was to explore the association between the routine lipid metabolism and adipose tissue distribution indicators and CKD prognosis. More lipid measures have emerged along with recent technological developments, however our study did not include the new measurements which might affect CKD prognosis. Further research on these indicators is necessary. Second, we did not collect other potential confounders which could also have affected the prognosis of patients with CKD, for example, physical activity. Third, we discussed the relationship between the baseline exposures and prognosis, but did not consider the effects of their dynamic changes on CKD prognosis. Fourth, there might have been a competitive risk of cardiovascular outcomes in this study. Finally, a single center cohort study is insufficient to demonstrate a causal relationship between fat-related indicators and composite outcomes, and thus the generalizability of the study findings is limited.

### Electronic supplementary material

Below is the link to the electronic supplementary material.

Supplementary Material 1

Supplementary Material 2

Supplementary Material 3

Supplementary Material 4

Supplementary Material 5

Supplementary Material 6

Supplementary Material 7

## Data Availability

Data described in the manuscript and the analytic code will be made available upon reasonable request to the corresponding author.
