# Supplementary material for: Lipid parameters, adipose tissue distribution and prognosis prediction in chronic kidney Disease patients
Source: Lipids Health Dis. 2024 Jan 8;23:5. doi: 10.1186/s12944-024-02004-4 (PMC10773091; doi:10.1186/s12944-024-02004-4)
Supplement: Supplementary file 1 — Supplementary Material 1 [file 12944_2024_2004_MOESM1_ESM.docx]

**Supplement file 1 Percentage of missing data**

| Variables | Proportion of missing data | Variables | Proportion of missing data |
| --- | --- | --- | --- |
| Gender | 0.00% | ALB, g/L | 4.53% |
| Marriage | 2.14% | eGFR, ml/min/1.73 m2 | 0.00% |
| Alcohol Consumption | 0.00% | K+, mmol/L | 14.11% |
| Current Smoker | 0.00% | Na+, mmol/L | 14.23% |
| Education | 0.25% | Ca, mmol/L | 18.01% |
| Without self-care capacity | 0.25% | P, mmol/L | 18.77% |
| Protopathy | 0.00% | UPCR, mg/g | 18.39% |
| Hypertension management | 0.00% | TG, mmol/L | 0.00% |
| Glu management | 0.00% | TC, mmol/L | 0.00% |
| UA management | 0.38% | HDL-C, mmol/L | 0.00% |
| With history of cardiovascular disease | 0.00% | LDL-C, mmol/L | 0.00% |
| ACEI/ARB | 0.00% | TSKF, cm | 0.00% |
| Other antihypertensive drugs | 0.00% | MUAC, cm | 0.00% |
| Hypoglycemic agents | 0.00% | BMI, kg/m2 | 0.00% |
| Urate-lowering drugs | 0.00% | BFM, kg | 0.00% |
| Lipid-lowering drugs | 0.00% | FMI, % | 0.00% |
| Folic acid tablets | 0.00% | PBF, % | 0.00% |
| Polysaccharide iron | 0.00% | VFA, cm2 | 0.00% |
| EPO | 0.00% | TBW, kg/L | 0.00% |
| Age | 0.00% | ICW, kg/L | 0.00% |
| Hb, g/L | 3.90% | ECW, kg/L | 0.00% |
| TCO2, mmol/L | 1.51% | FFM, kg | 0.00% |
| UA, μmmol/L | 0.63% | FFMI, % | 0.00% |
| Urea, mmol/L | 0.00% |  |  |

Note:Angiotensin converting enzyme, ACE; Angiotensin receptor blocker, ARB; Uric acid, UA; Erythropoietin, EPO; Hemoglobin, Hb; Triglyceride, TG; total cholesterol, TC; high-density lipoprotein cholesterol,HDL-C; low-density lipoprotein cholesterol, LDL-C; Total carbon dioxide, TCO2; albumin, ALB; Urine protein-to-creatinine, UPCR; Body Mass Index, BMI; triceps skinfold thickness, TSKF; mid-arm circumference, MUAC; Body Fat Mass, BFM; Fat Mass Index, FMI; Percent Body Fat, PBF; Visceral Fat Area, VFA; Total Body Water, TBW; Intracellular Water, ICW; Extracellular Water, ECW; Fat Free Mass, FFM; Fat Free Mass Index, FFMI.
