# Supplementary material for: Lipid parameters, adipose tissue distribution and prognosis prediction in chronic kidney Disease patients
Source: Lipids Health Dis. 2024 Jan 8;23:5. doi: 10.1186/s12944-024-02004-4 (PMC10773091; doi:10.1186/s12944-024-02004-4)
Supplement: Supplementary file 2 — Supplementary Material 2 [file 12944_2024_2004_MOESM2_ESM.docx]

**Supplement file 2 Schoenfeld residuals**

| Variables | chisq | *P* for PH |
| --- | --- | --- |
| TSKF | 0.934 | 0.330 |
| MUAC | 0.678 | 0.410 |
| BMI | 0.589 | 0.440 |
| BFM | 3.000 | 0.083 |
| PBF | 5.830 | 0.016 |
| VFA | 3.370 | 0.066 |
| FMI | 3.800 | 0.051 |
| TG | 0.225 | 0.640 |
| TC | 5.700 | 0.017 |
| HDL-C | 0.960 | 0.330 |
| LDL-C | 9.230 | 0.002 |
