# Supplementary material for: Lipid parameters, adipose tissue distribution and prognosis prediction in chronic kidney Disease patients
Source: Lipids Health Dis. 2024 Jan 8;23:5. doi: 10.1186/s12944-024-02004-4 (PMC10773091; doi:10.1186/s12944-024-02004-4)
Supplement: Supplementary file 3 — Supplementary Material 3 [file 12944_2024_2004_MOESM3_ESM.docx]

**Supplement file 3 Univariate Poisson Regression**

Table1 Univariate Poisson Regression for Categorical Variables

| Variables |  | RRs[95%CI] | *P* |
| --- | --- | --- | --- |
| Gender | Male | Ref |  |
|  | Female | 0.886[0.692,1.136] | 0.341 |
| Marrial status | Unmarried | Ref |  |
|  | Married | 0.511[0.327,0.799] | 0.003 |
| Alcohol consumption | No | Ref |  |
|  | Yes | 0.617[0.198,1.927] | 0.406 |
| Current smoker | No | Ref |  |
|  | Yes | 1.165[0.765,1.774] | 0.476 |
| Education |  |  |  |
| Elementary school |  | Ref |  |
| Junior high school | Dataset1 | 0.951[0.643,1.407] | 0.801 |
|  | Dataset2 | 0.946[0.640,1.400] | 0.783 |
|  | Dataset3 | 0.946[0.640,1.400] | 0.783 |
|  | Dataset4 | 0.951[0.643,1.407] | 0.801 |
|  | Dataset5 | 0.951[0.643,1.407] | 0.801 |
| High school | Dataset1 | 0.829[0.558,1.230] | 0.351 |
|  | Dataset2 | 0.829[0.558,1.230] | 0.351 |
|  | Dataset3 | 0.829[0.558,1.230] | 0.351 |
|  | Dataset4 | 0.825[0.556,1.225] | 0.340 |
|  | Dataset5 | 0.829[0.558,1.230] | 0.351 |
| College degree or above | Dataset1 | 1.124[0.751,1.680] | 0.571 |
|  | Dataset2 | 1.131[0.756,1.691] | 0.549 |
|  | Dataset3 | 1.131[0.756,1.691] | 0.549 |
|  | Dataset4 | 1.131[0.756,1.691] | 0.549 |
|  | Dataset5 | 1.124[0.751,1.680] | 0.571 |
| Without self-care capacity | No | Ref |  |
|  | Yes | 0.764[0.479,1.220] | 0.260 |
| Protopathy |  |  |  |
| Primary glomerular disease |  | Ref |  |
| Hypertensive nephropathy |  | 0.993[0.556,1.773] | 0.981 |
| Diabetic nephropathy |  | 1.002[0.668,1.504] | 0.991 |
| Others |  | 0.769[0.522,1.133] | 0.184 |
| Unknown |  | 0.808[0.600,1.086] | 0.158 |
| Comorbidity |  |  |  |
| Hypertension with normal BP |  | Ref |  |
| Hypertension with abnormal BP |  | 0.789[0.577,1.078] | 0.136 |
| Hypertension with without regular assessment |  | 1.247[0.826,1.881] | 0.293 |
| Non-hypertension |  | 0.745[0.499,1.114] | 0.151 |
| Diabetes with normal Glu |  | Ref |  |
| Diabetes with abnormal Glu |  | 1.211[0.781,1.878] | 0.391 |
| Diabetes without regular assessment |  | 0.859[0.487,1.515] | 0.599 |
| Non-diabetes |  | 1.072[0.757,1.517] | 0.696 |
| Hyperuricemia with normal uric acid |  | Ref |  |
| Hyperuricemia with anormal uric acid | Dataset1 | 0.997[0.737,1.350] | 0.986 |
|  | Dataset2 | 1.001[0.740,1.354] | 0.994 |
|  | Dataset3 | 0.995[0.735,1.346] | 0.972 |
|  | Dataset4 | 0.997[0.736,1.349] | 0.982 |
|  | Dataset5 | 0.997[0.736,1.349] | 0.982 |
| Non-hyperuricemia | Dataset1 | 0.923[0.654,1.304] | 0.651 |
|  | Dataset2 | 0.916[0.648,1.295] | 0.619 |
|  | Dataset3 | 0.926[0.656,1.308] | 0.663 |
|  | Dataset4 | 0.923[0.653,1.303] | 0.649 |
|  | Dataset5 | 0.923[0.653,1.303] | 0.649 |
| With history of cardiovascular disease | No | Ref |  |
|  | Yes | 0.894[0.656,1.217] | 0.476 |
| Medication |  | 0.000 |  |
| ACEI/ARB | No | Ref |  |
|  | Yes | 0.798[0.620,1.027] | 0.080 |
| Other antihypertensive drugs | No | Ref |  |
|  | Yes | 1.239[0.966,1.589] | 0.092 |
| Hypoglycemic agents | No | Ref |  |
|  | Yes | 1.024[0.756,1.385] | 0.879 |
| Urate-lowering drugs | No | Ref |  |
|  | Yes | 1.034[0.809,1.322] | 0.791 |
| Lipid-lowering drugs | No | Ref |  |
|  | Yes | 1.025[0.791,1.327] | 0.853 |
| Folic acid tablets | No | Ref |  |
|  | Yes | 1.276[0.866,1.878] | 0.217 |
| Polysaccharide iron | No | Ref |  |
|  | Yes | 1.093[0.777,1.537] | 0.611 |
| EPO | No | Ref |  |
|  | Yes | 1.273[0.920,1.763] | 0.146 |

Note:Primary Glomerulonephritides included chronic nephritis, nephropathy syndrome and IgA nephropathy.Other secondary nephrosis included systemic lupus erythematosus nephritis, Henoch-Schonlein purpura,Hepatitis B virus-associated nephritis and obstructive nephropathy, etc. Blood Pressure, BP; Angiotensin converting enzyme, ACE; Angiotensin receptor blocker, ARB; Uric acid, UA; Erythropoietin, EPO.

Table2 Univariate Poisson Regression for Continuous Variables

| Variables |  | RRs[95%CI] | *P* |
| --- | --- | --- | --- |
| Age, year |  | 0.981[0.973,0.990] | 0.000 |
| Hb, g/L | Dataset1 | 0.967[0.961,0.973] | 0.000 |
|  | Dataset2 | 0.966[0.960,0.971] | 0.000 |
|  | Dataset3 | 0.966[0.960,0.972] | 0.000 |
|  | Dataset4 | 0.966[0.960,0.972] | 0.000 |
|  | Dataset5 | 0.966[0.960,0.971] | 0.000 |
| TCO2, mmol/L | Dataset1 | 0.862[0.827,0.899] | 0.000 |
|  | Dataset2 | 0.862[0.826,0.898] | 0.000 |
|  | Dataset3 | 0.860[0.825,0.897] | 0.000 |
|  | Dataset4 | 0.862[0.827,0.899] | 0.000 |
|  | Dataset5 | 0.860[0.825,0.897] | 0.000 |
| UA, mmol/L | Dataset1 | 1.001[0.999,1.002] | 0.262 |
|  | Dataset2 | 1.001[1.000,1.002] | 0.248 |
|  | Dataset3 | 1.001[0.999,1.002] | 0.278 |
|  | Dataset4 | 1.001[1.000,1.002] | 0.239 |
|  | Dataset5 | 1.001[0.999,1.002] | 0.265 |
| Urea, mmol/L |  | 1.134[1.117,1.150] | 0.000 |
| ALB, g/L | Dataset1 | 0.939[0.921,0.957] | 0.000 |
|  | Dataset2 | 0.939[0.921,0.958] | 0.000 |
|  | Dataset3 | 0.940[0.922,0.959] | 0.000 |
|  | Dataset4 | 0.938[0.920,0.957] | 0.000 |
|  | Dataset5 | 0.940[0.922,0.958] | 0.000 |
| UPCR, mg/g | Dataset1 | 1.252[1.209,1.297] | 0.000 |
|  | Dataset2 | 1.264[1.221,1.308] | 0.000 |
|  | Dataset3 | 1.239[1.197,1.282] | 0.000 |
|  | Dataset4 | 1.264[1.222,1.308] | 0.000 |
|  | Dataset5 | 1.271[1.228,1.314] | 0.000 |
| eGFR, ml/min/1.73 m^2^ |  | 0.932[0.924,0.941] | 0.000 |
| K^+^, mmol/L | Dataset1 | 1.900[1.495,2.414] | 0.000 |
|  | Dataset2 | 1.908[1.502,2.422] | 0.000 |
|  | Dataset3 | 1.820[1.433,2.312] | 0.000 |
|  | Dataset4 | 1.796[1.421,2.270] | 0.000 |
|  | Dataset5 | 1.950[1.535,2.478] | 0.000 |
| Na^+^, mmol/L | Dataset1 | 0.913[0.865,0.963] | 0.001 |
|  | Dataset2 | 0.910[0.864,0.958] | 0.000 |
|  | Dataset3 | 0.920[0.873,0.969] | 0.002 |
|  | Dataset4 | 0.911[0.864,0.961] | 0.001 |
|  | Dataset5 | 0.891[0.845,0.938] | 0.000 |
| Ca^2+^, mmol/L | Dataset1 | 0.045[0.023,0.085] | 0.000 |
|  | Dataset2 | 0.048[0.025,0.093] | 0.000 |
|  | Dataset3 | 0.052[0.026,0.101] | 0.000 |
|  | Dataset4 | 0.053[0.027,0.104] | 0.000 |
|  | Dataset5 | 0.049[0.025,0.095] | 0.000 |
| P, mmol/L | Dataset1 | 18.116[12.599,26.050] | 0.000 |
|  | Dataset2 | 17.878[12.492,25.585] | 0.000 |
|  | Dataset3 | 17.121[11.899,24.636] | 0.000 |
|  | Dataset4 | 17.707[12.338,25.414] | 0.000 |
|  | Dataset5 | 17.715[12.306,25.501] | 0.000 |
| TG, mmol/L |  | 1.035[0.940,1.140] | 0.481 |
| TC, mmol/L |  | 1.125[1.032,1.227] | 0.008 |
| HDL-C, mmol/L |  | 1.162[0.878,1.538] | 0.293 |
| LDL-C, mmol/L |  | 1.100[0.992,1.219] | 0.071 |
| TSKF, cm |  | 0.729[0.609,0.874] | 0.001 |
| MUAC, cm |  | 0.942[0.907,0.978] | 0.002 |
| BMI, kg/m^2^ |  | 0.939[0.906,0.974] | 0.001 |
| BFM, kg |  | 0.937[0.917,0.956] | 0.000 |
| PBF, % |  | 0.951[0.936,0.965] | 0.000 |
| VFA, cm^2^ |  | 0.989[0.985,0.993] | 0.000 |
| FMI, % |  | 0.860[0.817,0.906] | 0.000 |
| FFMI, % |  | 1.054[0.992,1.120] | 0.091 |
| FFM, kg |  | 1.007[0.992,1.023] | 0.337 |
| TBW, kg/L |  | 1.011[0.991,1.031] | 0.297 |
| ICW, kg/L |  | 1.007[0.974,1.041] | 0.672 |
| ECW, kg/L |  | 1.053[1.001,1.108] | 0.047 |

Note:Hemoglobin, Hb; Triglyceride, TG; total cholesterol, TC; high-density lipoprotein cholesterol,HDL-C; low-density lipoprotein cholesterol, LDL-C; Total carbon dioxide, TCO2; K+; Na+; Ca2+; P; albumin, ALB; Urine protein-to-creatinine, UPCR; Body Mass Index, BMI; triceps skinfold thickness, TSKF; mid-arm circumference, MUAC; Body Fat Mass, BFM; Fat Mass Index, FMI; Percent Body Fat, PBF; Visceral Fat Area, VFA; Total Body Water, TBW; Intracellular Water, ICW; Extracellular Water, ECW; Fat Free Mass, FFM; Fat Free Mass Index, FFMI.
