# Supplementary material for: Lipid parameters, adipose tissue distribution and prognosis prediction in chronic kidney Disease patients
Source: Lipids Health Dis. 2024 Jan 8;23:5. doi: 10.1186/s12944-024-02004-4 (PMC10773091; doi:10.1186/s12944-024-02004-4)
Supplement: Supplementary file 4 — Supplementary Material 4 [file 12944_2024_2004_MOESM4_ESM.docx]

**Supplement files 6 Multivariable-adjusted Poisson Models**

Table1 Multivariable-adjusted Models of TC

|  | Dataset 1 |  |  | Dataset 2 |  |  | Dataset 3 |  |  | Dataset 4 |  |  | Dataset 5 |  |  | Pooled |  |  |
| --- | --- | --- | --- | --- | --- | --- | --- | --- | --- | --- | --- | --- | --- | --- | --- | --- | --- | --- |
| Variables | RRs[95%CI] | *P* | *P* for interaction | RRs[95%CI] | *P* | *P* for interaction | RRs[95%CI] | *P* | *P* for interaction | RRs[95%CI] | *P* | *P* for interaction | RRs[95%CI] | *P* | *P* for interaction | RRs[95%CI] | *P* | *P* for interaction |
| Age |  |  | 0.846 |  |  | 0.771 |  |  | 0.878 |  |  | 0.571 |  |  | 0.602 |  |  | 0.828 |
| ＜58.8 | 1.115[0.969,1.283] | 0.130 |  | 1.156[1.013,1.320] | 0.031 |  | 1.178[1.037,1.340] | 0.012 |  | 1.090[0.944,1.260] | 0.241 |  | 1.126[0.982,1.290] | 0.089 |  | 1.133[1.122,1.144] | 0.107 |  |
| ≥58.8 | 1.122[0.973,1.293] | 0.113 |  | 1.070[0.929,1.230] | 0.348 |  | 1.140[0.990,1.310] | 0.068 |  | 1.140[0.991,1.300] | 0.067 |  | 1.150[1.000,1.320] | 0.049 |  | 1.123[1.112,1.134] | 0.138 |  |
| Gender |  |  | 0.045 |  |  | 0.038 |  |  | 0.071 |  |  | 0.053 |  |  | 0.066 |  |  | 0.055 |
| Male | 1.078[0.946,1.23] | 0.259 |  | 1.060[0.935,1.210] | 0.347 |  | 1.120[0.986,1.270] | 0.083 |  | 1.070[0.940,1.230] | 0.292 |  | 1.100[0.970,1.250] | 0.135 |  | 1.087[1.078,1.095] | 0.230 |  |
| Female | 1.222[1.041,1.434] | 0.014 |  | 1.227[1.060,1.430] | 0.008 |  | 1.270[1.099,1.470] | 0.001 |  | 1.190[1.020,1.390] | 0.030 |  | 1.240[1.058,1.440] | 0.008 |  | 1.229[1.214,1.243] | 0.013 |  |
| CKD stage |  |  |  |  |  |  |  |  |  |  |  |  |  |  |  |  |  |  |
| Stage 3 | 1.066[0.892,1.273] | 0.481 |  | 1.110[0.937,1.320] | 0.222 |  | 1.190[1.013,1.390] | 0.034 |  | 1.050[0.867,1.260] | 0.641 |  | 1.110[0.926,1.330] | 0.263 |  | 1.103[1.086,1.120] | 0.347 |  |
| Stage 4 | 1.067[0.857,1.328] | 0.563 | 0.200 | 1.100[0.895,1.360] | 0.359 | 0.266 | 1.150[0.934,1.406] | 0.191 | 0.216 | 1.060[0.852,1.320] | 0.593 | 0.102 | 1.120[0.911,1.380] | 0.279 | 0.135 | 1.100[1.074,1.126] | 0.405 | 0.186 |
| Stage 5 | 1.165[1.002,1.355] | 0.046 | 0.808 | 1.160[0.997,1.340] | 0.055 | 0.667 | 1.200[1.030,1.390] | 0.016 | 0.768 | 1.190[1.030,1.380] | 0.022 | 0.806 | 1.190[1.020,1.380] | 0.023 | 0.935 | 1.179[1.166,1.193] | 0.034 | 0.798 |
| Hypertension |  |  | 0.734 |  |  | 0.971 |  |  | 0.888 |  |  | 0.781 |  |  | 0.843 |  |  | 0.855 |
| With hypertension | 1.148[1.032,1.276] | 0.011 |  | 1.150[1.035,1.270] | 0.009 |  | 1.202[1.090,1.330] | 0.000 |  | 1.140[1.023,1.270] | 0.018 |  | 1.180[1.060,1.300] | 0.002 |  | 1.162[1.155,1.169] | 0.010 |  |
| Non-hypertension | 0.974[0.695,1.364] | 0.878 |  | 1.040[0.770,1.400] | 0.799 |  | 1.020[0.742,1.400] | 0.904 |  | 1.000[0.739,1.350] | 0.998 |  | 0.995[0.728,1.360] | 0.974 |  | 1.006[0.956,1.059] | 0.973 |  |
| Diabetes |  |  | 0.465 |  |  | 0.710 |  |  | 0.492 |  |  | 0.277 |  |  | 0.386 |  |  | 0.471 |
| With diabetes | 1.165[1,1.357] | 0.050 |  | 1.120[0.966,1.300] | 0.135 |  | 1.200[1.030,1.390] | 0.016 |  | 1.180[1.014,1.370] | 0.032 |  | 1.180[1.013,1.370] | 0.033 |  | 1.168[1.154,1.181] | 0.057 |  |
| Non-diabetes | 1.138[0.989,1.309] | 0.072 |  | 1.152[1.006,1.320] | 0.040 |  | 1.200[1.057,1.360] | 0.005 |  | 1.109[0.962,1.280] | 0.153 |  | 1.163[1.013,1.340] | 0.033 |  | 1.153[1.141,1.164] | 0.065 |  |
| Lipid-lowering drugs |  |  | 0.115 |  |  | 0.257 |  |  | 0.187 |  |  | 0.114 |  |  | 0.106 |  |  | 0.161 |
| YES | 1.072[0.895,1.284] | 0.452 |  | 1.100[0.933,1.300] | 0.255 |  | 1.150[0.980,1.350] | 0.086 |  | 1.080[0.903,1.290] | 0.402 |  | 1.120[0.943,1.330] | 0.200 |  | 1.103[1.086,1.120] | 0.288 |  |
| No | 1.190[1.052,1.345] | 0.006 |  | 1.162[1.030,1.310] | 0.012 |  | 1.217[1.081,1.370] | 0.001 |  | 1.178[1.040,1.330] | 0.010 |  | 1.203[1.065,1.360] | 0.003 |  | 1.190[1.181,1.199] | 0.008 |  |

Table2 Multivariable-adjusted Models of PBF

|  | Dataset 1 |  |  | Dataset 2 |  |  | Dataset 3 |  |  | Dataset 4 |  |  | Dataset 5 |  |  | Pooled |  |  |
| --- | --- | --- | --- | --- | --- | --- | --- | --- | --- | --- | --- | --- | --- | --- | --- | --- | --- | --- |
| Variables | RRs[95%CI] | *P* | *P* for interaction | RRs[95%CI] | *P* | *P* for interaction | RRs[95%CI] | *P* | *P* for interaction | RRs[95%CI] | *P* | *P* for interaction | RRs[95%CI] | *P* | *P* for interaction | RRs[95%CI] | *P* | *P* for interaction |
| Age |  |  | 0.575 |  |  | 0.662 |  |  | 0.723 |  |  | 0.618 |  |  | 0.546 |  |  | 0.620 |
| ＜58.8 | 0.970[0.949,0.991] | 0.006 |  | 0.972[0.950,0.995] | 0.016 |  | 0.968[0.947,0.989] | 0.003 |  | 0.970[0.950,0.991] | 0.006 |  | 0.968[0.947,0.988] | 0.002 |  | 0.969[0.948,0.992] | 0.007 |  |
| ≥58.8 | 0.989[0.966,1.013] | 0.374 |  | 0.980[0.959,1.000] | 0.066 |  | 0.985[0.962,1.010] | 0.199 |  | 0.988[0.964,1.010] | 0.324 |  | 0.987[0.964,1.010] | 0.284 |  | 0.986[0.962,1.01] | 0.253 |  |
| Gender |  |  | 0.245 |  |  | 0.295 |  |  | 0.188 |  |  | 0.299 |  |  | 0.323 |  |  | 0.291 |
| Male | 0.972[0.951,0.994] | 0.014 |  | 0.968[0.947,0.989] | 0.003 |  | 0.970[0.948,0.991] | 0.006 |  | 0.974[0.952,0.996] | 0.020 |  | 0.971[0.950,0.993] | 0.009 |  | 0.970[0.949,0.993] | 0.009 |  |
| Female | 0.983[0.962,1.005] | 0.126 |  | 0.985[0.961,1.010] | 0.219 |  | 0.981[0.960,1.000] | 0.091 |  | 0.984[0.963,1.010] | 0.148 |  | 0.981[0.961,1.000] | 0.086 |  | 0.983[0.962,1.005] | 0.131 |  |
| CKD stage |  |  |  |  |  |  |  |  |  |  |  |  |  |  |  |  |  |  |
| Stage 3 | 0.982[0.949,1.016] | 0.289 |  | 0.969[0.936,1.000] | 0.080 |  | 0.974[0.941,1.010] | 0.142 |  | 0.972[0.939,1.010] | 0.109 |  | 0.970[0.937,1.000] | 0.080 |  | 0.973[0.939,1.009] | 0.147 |  |
| Stage 4 | 0.971[0.947,0.996] | 0.025 | 0.608 | 0.967[0.943,0.992] | 0.010 | 0.675 | 0.966[0.942,0.991] | 0.007 | 0.893 | 0.971[0.946,0.996] | 0.023 | 0.563 | 0.966[0.942,0.990] | 0.006 | 0.690 | 0.969[0.943,0.994] | 0.015 | 0.693 |
| Stage 5 | 0.991[0.965,1.019] | 0.528 | 0.096 | 0.99[0.963,1.020] | 0.476 | 0.081 | 0.990[0.964,1.020] | 0.477 | 0.180 | 0.994[0.967,1.020] | 0.655 | 0.069 | 0.995[0.968,1.020] | 0.714 | 0.083 | 0.992[0.965,1.02] | 0.574 | 0.102 |
| Hypertension |  |  | 0.257 |  |  | 0.241 |  |  | 0.284 |  |  | 0.186 |  |  | 0.197 |  |  | 0.250 |
| With hypertension | 0.973[0.957,0.990] | 0.002 |  | 0.970[0.954,0.987] | 0.000 |  | 0.970[0.954,0.987] | 0.000 |  | 0.972[0.956,0.989] | 0.001 |  | 0.970[0.954,0.987] | 0.000 |  | 0.971[0.954,0.989] | 0.001 |  |
| Non-hypertension | 0.999[0.961,1.038] | 0.957 |  | 0.998[0.960,1.040] | 0.923 |  | 0.991[0.954,1.030] | 0.630 |  | 1.010[0.965,1.050] | 0.801 |  | 1.000[0.964,1.040] | 0.936 |  | 0.999[0.959,1.041] | 0.852 |  |
| Diabetes |  |  | 0.186 |  |  | 0.265 |  |  | 0.322 |  |  | 0.177 |  |  | 0.197 |  |  | 0.231 |
| With diabetes | 0.988[0.963,1.013] | 0.332 |  | 0.987[0.962,1.010] | 0.292 |  | 0.982[0.958,1.010] | 0.131 |  | 0.989[0.964,1.010] | 0.380 |  | 0.987[0.963,1.010] | 0.281 |  | 0.986[0.962,1.012] | 0.291 |  |
| Non-diabetes | 0.969[0.950,0.989] | 0.002 |  | 0.968[0.949,0.988] | 0.001 |  | 0.968[0.948,0.987] | 0.001 |  | 0.969[0.950,0.989] | 0.002 |  | 0.966[0.947,0.986] | 0.001 |  | 0.969[0.949,0.988] | 0.001 |  |
| Lipid-lowering drugs |  |  | 0.639 |  |  | 0.585 |  |  | 0.950 |  |  | 0.536 |  |  | 0.518 |  |  | 0.646 |
| YES | 0.967[0.938,0.998] | 0.034 |  | 0.968[0.938,0.998] | 0.038 |  | 0.968[0.938,0.998] | 0.039 |  | 0.968[0.939,0.998] | 0.039 |  | 0.966[0.936,0.996] | 0.026 |  | 0.968[0.937,0.998] | 0.037 |  |
| No | 0.979[0.961,0.997] | 0.021 |  | 0.977[0.959,0.995] | 0.012 |  | 0.976[0.958,0.994] | 0.008 |  | 0.980[0.961,0.998] | 0.029 |  | 0.979[0.961,0.997] | 0.024 |  | 0.977[0.96,0.995] | 0.014 |  |
