# Supplementary material for: Lipid parameters, adipose tissue distribution and prognosis prediction in chronic kidney Disease patients
Source: Lipids Health Dis. 2024 Jan 8;23:5. doi: 10.1186/s12944-024-02004-4 (PMC10773091; doi:10.1186/s12944-024-02004-4)
Supplement: Supplementary file 5 — Supplementary Material 5 [file 12944_2024_2004_MOESM5_ESM.docx]

Supplement file 7 Sensitivity Analysis

Table 1 Multivariable-adjusted Poisson Models of Sensitivity Analysis

|  | Model 1 |  | Model 3 |  |
| --- | --- | --- | --- | --- |
| Variables | RRs[95%CI] | *P* | RRs[95%CI] | *P* |
| Age, year | 0.98[0.969,0.992] | 0.001 | 0.982[0.971,0.994] | 0.003 |
| Hb, g/L | 0.996[0.987,1.006] | 0.454 | 0.996[0.987,1.005] | 0.392 |
| Urea, mmol/L | 1.030[1.003,1.065] | 0.031 | 1.030[1.003,1.065] | 0.032 |
| UPCR, mg/g | 1.220[1.153,1.288] | 0.000 | 1.210[1.145,1.281] | 0.000 |
| eGFR, ml/min/1.73 m^2^ | 0.961[0.947,0.976] | 0.000 | 0.961[0.947,0.976] | 0.000 |
| K^+^, mmol/L | 1.140[0.872,1.499] | 0.334 | -- | -- |
| Ca^2+^, mmol/L | 0.606[0.199,1.848] | 0.379 | 0.718[0.236,2.185] | 0.560 |
| P, mmol/L | 1.730[1.089,2.754] | 0.020 | 1.740[1.101,2.752] | 0.018 |
| PBF, % | -- | -- | 0.990[0.972,1.009] | 0.302 |

Notes: Poisson regression with Lasso method. Model 1: Age, Hb, TCO2, Urea, ALB, UPCR, eGFR, K^+^, Ca^2+^, P, ECW; Model 2: Model 1+TSKF; Model 3: Model 1+BFM, PBF, VFA, FMI, FFMI; Model 4:Model 1+TSKF, BFM,PBF,VFA,FMI. The Model 1 and Model 2 had same results. The Model 3 and Model 4 had same results.

Table 2 Graphs of Lasso regression

|  | Sensitivity analysis | |
| --- | --- | --- |
| Model1 | 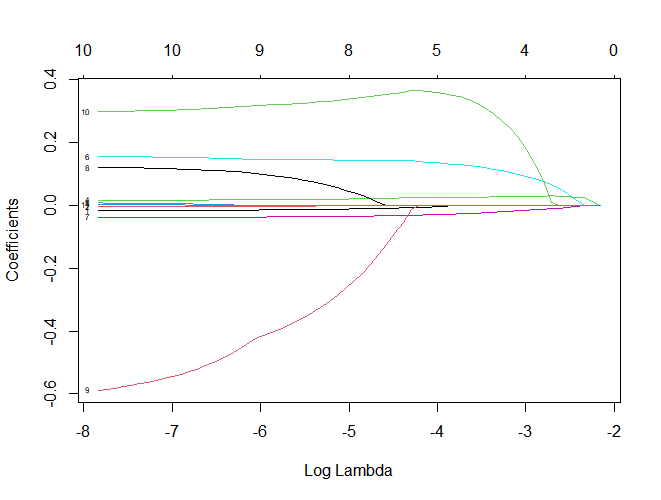 | 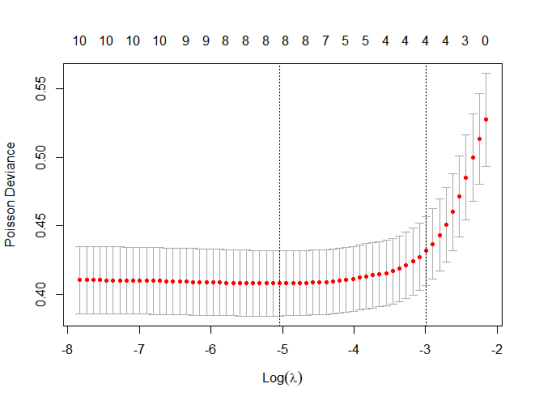 |
| Model2 | 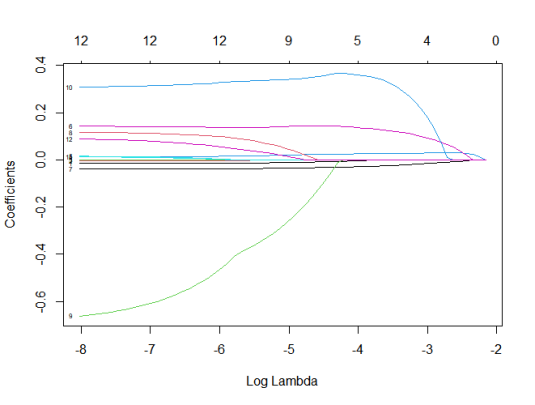 | 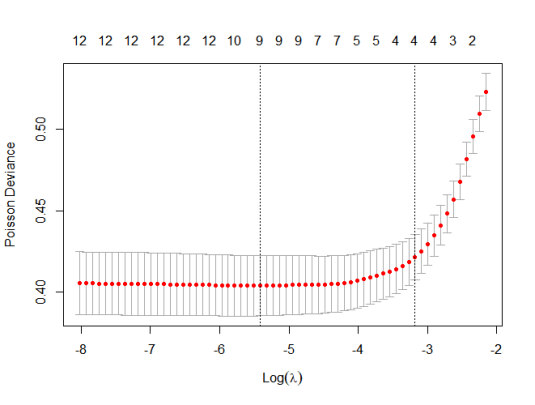 |
| Model3 | 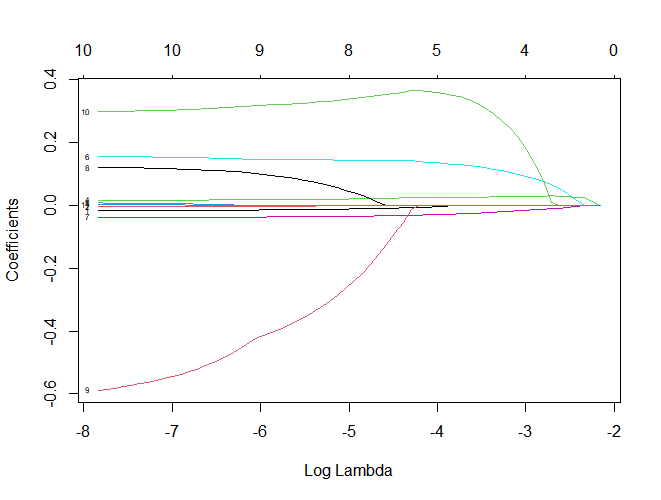 | 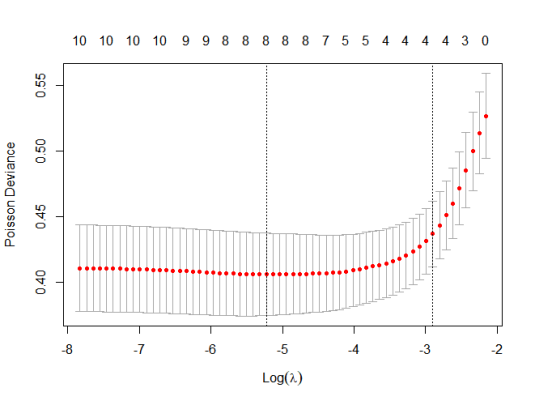 |
| Model4 | 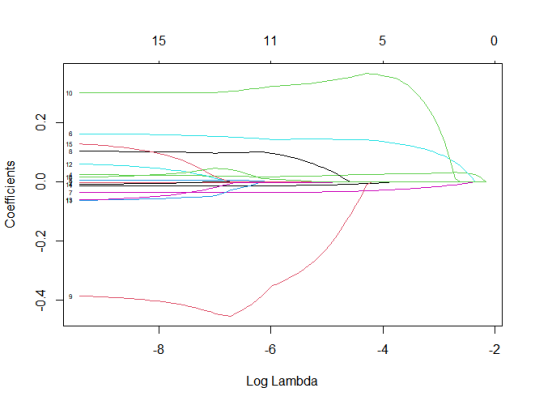 | 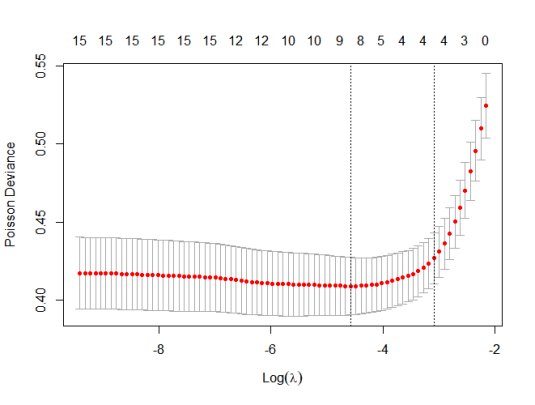 |
| Model5 | 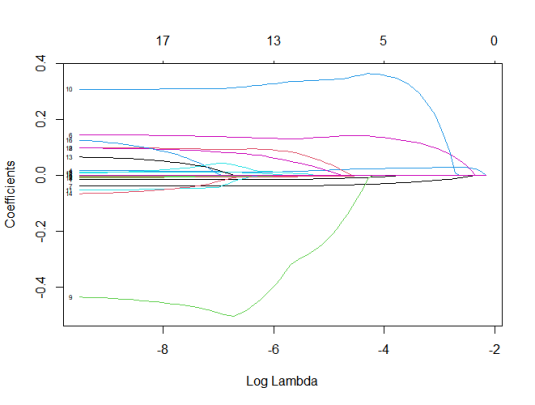 | 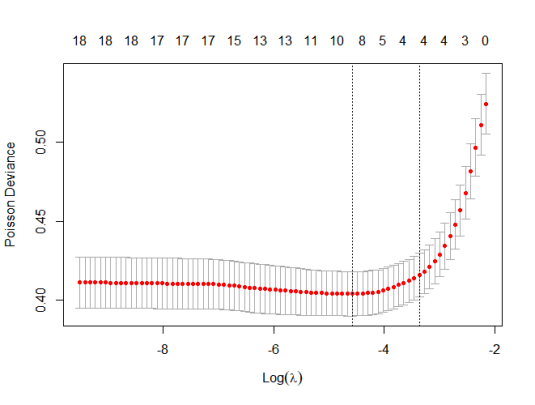 |

Table 3 Models Comparison of Sensitivity Analysis

|  | Model 1 | Model 3 |
| --- | --- | --- |
| AIC | 1192.8 | 1192.6 |
| AUC | 0.685[0.642,0.728] | 0.685[0.642,0.728] |
| *P* for ROC | 0.918 | Ref |
| NRI | 0.009[-0.049,0.050] | Ref |

Notes: Akaike information criterion, AIC; the area under the receiver operating curve, AUC; receiver operating curve, ROC; net reclassification index, NRI.
