# Supplementary material for: Lipid parameters, adipose tissue distribution and prognosis prediction in chronic kidney Disease patients
Source: Lipids Health Dis. 2024 Jan 8;23:5. doi: 10.1186/s12944-024-02004-4 (PMC10773091; doi:10.1186/s12944-024-02004-4)
Supplement: Supplementary file 6 — Supplementary Material 6 [file 12944_2024_2004_MOESM6_ESM.docx]

**Supplement files 4 Multivariable-adjusted Poisson Models**

Table1 Multivariable-adjusted Models with Dataset1

|  | Model1 |  | Model 2 |  | Model 3 |  | Model 4 |  | Model 5 |  |
| --- | --- | --- | --- | --- | --- | --- | --- | --- | --- | --- |
| Variables | RRs[95%CI] | *P* | RRs[95%CI] | *P* | RRs[95%CI] | *P* | RRs[95%CI] | *P* | RRs[95%CI] | *P* |
| TC, mmol/L | -- | -- | 1.130[1.025,1.250] | 0.014 | -- | -- | -- | -- | 1.150[1.044,1.270] | 0.005 |
| BMI, kg/m^2^ | -- | -- | -- | -- | 0.973[0.938,1.009] | 0.140 | -- | -- | -- | -- |
| PBF, % | -- | -- | -- | -- | -- | -- | 0.979[0.963,0.994] | 0.007 | 0.976[0.961,0.992] | 0.003 |
| Age, year | 0.989[0.980,0.998] | 0.014 | 0.988[0.979,0.997] | 0.008 | 0.987[0.978,0.996] | 0.005 | 0.990[0.981,0.999] | 0.037 | 0.990[0.980,0.999] | 0.026 |
| Hb, g/L | 0.995[0.988,1.003] | 0.230 | 0.991[0.983,0.999] | 0.023 | 0.994[0.986,1.002] | 0.121 | 0.993[0.986,1.000] | 0.076 | 0.990[0.982,0.998] | 0.013 |
| TCO2, mmol/L | -- | -- |  |  |  |  | 0.993[0.954,1.030] | 0.745 | 0.988[0.948,1.030] | 0.549 |
| Urea, mmol/L | 1.016[0.989,1.044] | 0.254 | 1.020[0.993,1.050] | 0.154 | 1.030[1.001,1.057] | 0.043 | 1.020[0.995,1.050] | 0.111 | 1.020[0.988,1.040] | 0.258 |
| UPCR, mg/g | 1.231[1.177,1.287] | <0.000 | 1.160[1.104,1.230] | 0.000 | 1.200[1.151,1.261] | 0.000 | 1.200[1.143,1.260] | 0.000 | 1.150[1.087,1.210] | 0.000 |
| eGFR, ml/min/1.73 m^2^ | 0.957[0.945,0.969] | 0.000 | 0.956[0.944,0.968] | 0.000 | 0.960[0.948,0.972] | 0.000 | 0.958[0.947,0.970] | 0.000 | 0.956[0.944,0.968] | 0.000 |
| K^+^, mmol/L | 1.252[0.993,1.579] | 0.058 | 1.200[0.950,1.500] | 0.128 | 1.200[0.950,1.506] | 0.128 | 1.130[0.887,1.440] | 0.320 | 1.150[0.900,1.460] | 0.267 |
| Na^+^, mmol/L | 0.964[0.919,1.013] | 0.146 | 0.955[0.907,1.000] | 0.075 | -- | -- | 0.957[0.908,1.010] | 0.100 | 0.960[0.911,1.010] | 0.128 |
| Ca^2+^, mmol/L | -- | -- | 0.588[0.243,1.420] | 0.239 | 0.560[0.227,1.383] | 0.209 | 0.804[0.310,2.080] | 0.654 | 0.826[0.322,2.120] | 0.692 |
| P, mmol/L | 2.922[1.725,4.951] | 0.000 | 2.310[1.350,3.940] | 0.002 | 2.300[1.348,3.909] | 0.002 | 2.290[1.336,3.920] | 0.003 | 2.300[1.342,3.930] | 0.002 |

Table2 Multivariable-adjusted Models with Dataset2

|  | Model1 |  | Model 2 |  | Model 3 |  | Model 4 |  | lambda.1se2 |  | Model 5 |  |
| --- | --- | --- | --- | --- | --- | --- | --- | --- | --- | --- | --- | --- |
| Variables | RRs[95%CI] | *P* | RRs[95%CI] | *P* | RRs[95%CI] | *P* | RRs[95%CI] | *P* | RRs[95%CI] | *P* | RRs[95%CI] | *P* |
| TC, mmol/L | -- | -- | 1.113[1.012,1.224] | 0.027 | -- | -- | -- | -- | -- | -- | 1.130[1.026,1.240] | 0.013 |
| BMI, kg/m^2^ | -- | -- | -- | -- | 0.973[0.937,1.010] | 0.145 | -- | -- | -- | -- | -- | -- |
| PBF, % | -- | -- | -- | -- | -- | -- | 0.976[0.962,0.991] | 0.002 | 0.973[0.958,0.987] | 0.000 | 0.975[0.960,0.990] | 0.001 |
| Age, year | 0.988[0.979,0.997] | 0.007 | 0.987[0.978,0.996] | 0.004 | 0.988[0.979,0.997] | 0.007 | 0.99[0.981,0.999] | 0.032 | -- | -- | 0.989[0.98,0.998] | 0.020 |
| Hb, g/L | 0.995[0.988,1.003] | 0.246 | 0.993[0.986,1.001] | 0.101 | 0.996[0.988,1.003] | 0.258 | 0.995[0.988,1.003] | 0.204 | -- | -- | 0.993[0.985,1.001] | 0.067 |
| TCO2, mmol/L | -- | -- | -- | -- | -- | -- | 0.991[0.952,1.032] | 0.668 | -- | -- | 0.987[0.948,1.028] | 0.537 |
| Urea, mmol/L | 1.020[0.991,1.047] | 0.180 | 1.016[0.988,1.044] | 0.261 | 1.020[0.992,1.048] | 0.134 | 1.020[0.989,1.043] | 0.258 | 1.020[0.997,1.050] | 0.086 | 1.010[0.985,1.039] | 0.402 |
| UPCR, mg/g | 1.230[1.172,1.289] | <0.000 | 1.188[1.123,1.257] | 0.000 | 1.240[1.182,1.294] | <0.000 | 1.220[1.163,1.276] | <0.000 | 1.220[1.173,1.277] | <0.000 | 1.170[1.110,1.240] | 0.000 |
| eGFR, ml/min/1.73 m^2^ | 0.958[0.946,0.969] | 0.000 | 0.956[0.944,0.968] | 0.000 | 0.958[0.947,0.970] | 0.000 | 0.958[0.946,0.969] | 0.000 | 0.957[0.945,0.968] | 0.000 | 0.956[0.944,0.968] | 0.000 |
| K^+^, mmol/L | 1.260[0.995,1.588] | 0.055 | 1.277[1.011,1.613] | 0.040 | 1.250[0.993,1.579] | 0.057 | 1.200[0.938,1.523] | 0.149 | -- | -- | 1.220[0.955,1.550] | 0.112 |
| Ca^2+^, mmol/L | 0.887[0.351,2.242] | 0.800 | 0.849[0.338,2.132] | 0.728 | -- | -- | -- | -- | -- | -- | -- | -- |
| P, mmol/L | 2.960[1.743,5.013] | 0.000 | 2.983[1.761,5.055] | 0.000 | 2.850[1.680,4.822] | 0.000 | 2.820[1.671,4.774] | 0.000 | 3.120[1.876,5.199] | 0.000 | 2.820[1.677,4.759] | 0.000 |

Table3 Multivariable-adjusted Models with Dataset3

|  | Model1 |  | Model 2 |  | Model 3 |  | Model 4 |  | Model 5 |  |
| --- | --- | --- | --- | --- | --- | --- | --- | --- | --- | --- |
| Variables | RRs[95%CI] | *P* | RRs[95%CI] | *P* | RRs[95%CI] | *P* | RRs[95%CI] | *P* | RRs[95%CI] | *P* |
| TC, mmol/L | -- | -- | 1.170[1.070,1.285] | 0.001 | -- | -- | -- | -- | 1.190[1.088,1.305] | 0.000 |
| BMI, kg/m^2^ | -- | -- | -- | -- | 0.976[0.940,1.013] | 0.197 | -- | -- | -- | -- |
| PBF, % | -- | -- | -- | -- | -- | -- | 0.978[0.982,1.000] | 0.006 | 0.975[0.960,0.990] | 0.001 |
| Age, year | 0.988[0.979,0.997] | 0.011 | 0.987[0.978,0.996] | 0.004 | 0.988[0.979,0.997] | 0.011 | 0.991[0.982,1.000] | 0.058 | 0.989[0.980,0.998] | 0.018 |
| Hb, g/L | 0.995[0.987,1.003] | 0.202 | 0.992[0.984,1.000] | 0.044 | 0.996[0.988,1.003] | 0.262 | 0.994[0.987,1.000] | 0.150 | 0.991[0.983,0.999] | 0.020 |
| TCO2, mmol/L | 0.988[0.950,1.028] | 0.564 | -- | -- | -- | -- | 0.985[0.947,1.030] | 0.462 | 0.980[0.942,1.020] | 0.334 |
| Urea, mmol/L | 1.010[0.987,1.042] | 0.306 | 1.010[0.984,1.038] | 0.420 | 1.020[0.989,1.043] | 0.247 | 1.010[0.985,1.040] | 0.384 | 1.010[0.981,1.035] | 0.559 |
| UPCR, mg/g | 1.180[1.127,1.235] | 0.000 | 1.140[1.086,1.199] | 0.000 | 1.190[1.133,1.241] | 0.000 | 1.170[1.119,1.230] | 0.000 | 1.130[1.070,1.183] | 0.000 |
| eGFR, ml/min/1.73 m^2^ | 0.955[0.944,0.967] | 0.000 | 0.953[0.941,0.964] | 0.000 | 0.955[0.944,0.967] | 0.000 | 0.955[0.943,0.967] | 0.000 | 0.953[0.941,0.965] | 0.000 |
| K^+^, mmol/L | 1.200[0.946,1.516] | 0.133 | 1.260[0.997,1.580] | 0.053 | 1.210[0.965,1.528] | 0.098 | 1.140[0.898,1.450] | 0.279 | 1.180[0.931,1.501] | 0.170 |
| Na^+^, mmol/L | -- | -- | -- | -- | -- | -- | 0.968[0.922,1.020] | 0.199 |  |  |
| Ca^2+^, mmol/L | 0.653[0.269,1.583] | 0.345 | 0.666[0.278,1.595] | 0.362 | 0.654[0.271,1.575] | 0.343 | 0.928[0.366,2.350] | 0.876 | -- | -- |
| P, mmol/L | 2.690[1.587,4.558] | 0.000 | 2.780[1.649,4.680] | 0.000 | 2.610[1.540,4.427] | 0.000 | 2.520[1.484,4.270] | 0.001 | 2.630[1.574,4.388] | 0.000 |

Table4 Multivariable-adjusted Models with Dataset4

|  | Model1 |  | Model 2 |  | Model 3 |  | Model 4 |  | Model 5 |  |
| --- | --- | --- | --- | --- | --- | --- | --- | --- | --- | --- |
| Variables | RRs[95%CI] | *P* | RRs[95%CI] | *P* | RRs[95%CI] | *P* | RRs[95%CI] | *P* | RRs[95%CI] | *P* |
| TC, mmol/L | -- | -- | 1.100[0.996,1.220] | 0.060 | -- | -- | -- | -- | 1.120[1.011,1.240] | 0.030 |
| TSKF, cm | -- | -- | -- | -- | 0.874[0.704,1.080] | 0.222 | -- | -- | -- | -- |
| BMI, kg/m^2^ | -- | -- | -- | -- | 0.993[0.953,1.030] | 0.730 | -- | -- | -- | -- |
| PBF, % | -- | -- | -- | -- | -- | -- | 0.979[0.964,0.995] | 0.008 | 0.978[0.963,0.993] | 0.004 |
| Age, year | 0.989[0.980,0.998] | 0.016 | 0.988[0.979,0.998] | 0.013 | 0.989[0.980,0.998] | 0.015 | 0.991[0.981,1.000] | 0.043 | 0.990[0.981,0.999] | 0.034 |
| Hb, g/L | 0.994[0.987,1.000] | 0.132 | 0.992[0.984,1.000] | 0.051 | 0.994[0.987,1.000] | 0.135 | 0.994[0.987,1.000] | 0.123 | 0.992[0.984,1.000] | 0.039 |
| TCO2, mmol/L | 0.996[0.957,1.040] | 0.844 | 0.993[0.955,1.030] | 0.728 |  |  | 0.991[0.952,1.030] | 0.658 | 0.987[0.948,1.030] | 0.512 |
| Urea, mmol/L | 1.030[1.004,1.060] | 0.025 | 1.030[0.998,1.050] | 0.071 | 1.030[1.003,1.060] | 0.028 | 1.030[1.001,1.060] | 0.042 | 1.020[0.994,1.050] | 0.130 |
| UPCR, mg/g | 1.240[1.187,1.300] | <0.000 | 1.200[1.141,1.270] | 0.000 | 1.250[1.193,1.310] | <0.000 | 1.230[1.174,1.290] | <0.000 | 1.190[1.122,1.250] | 0.000 |
| eGFR, ml/min/1.73 m^2^ | 0.961[0.949,0.973] | 0.000 | 0.959[0.946,0.971] | 0.000 | 0.962[0.950,0.974] | 0.000 | 0.960[0.948,0.972] | 0.000 | 0.958[0.945,0.970] | 0.000 |
| K^+^, mmol/L | 1.190[0.943,1.500] | 0.143 | 1.200[0.952,1.510] | 0.123 | 1.180[0.938,1.470] | 0.160 | 1.140[0.904,1.440] | 0.265 | 1.160[0.917,1.470] | 0.217 |
| Na^+^, mmol/L | 0.952[0.905,1.000] | 0.057 | 0.951[0.904,1.000] | 0.052 | 0.956[0.908,1.010] | 0.085 | 0.957[0.909,1.010] | 0.096 | 0.957[0.909,1.010] | 0.096 |
| Ca^2+^, mmol/L |  |  | -- | -- | -- | -- | -- | -- | -- | -- |
| P, mmol/L | 2.490[1.453,4.270] | 0.001 | 2.580[1.504,4.420] | 0.001 | 2.470[1.453,4.210] | 0.001 | 2.350[1.383,4.000] | 0.002 | 2.420[1.428,4.120] | 0.001 |

Table 5 Multivariable-adjusted Poisson Models with Dataset5

|  | Model1 | | Model 2 | | Model 3 | | Model 4 | | Model 5 | |
| --- | --- | --- | --- | --- | --- | --- | --- | --- | --- | --- |
| Variables | RRs[95%CI] | *P* | RRs[95%CI] | *P* | RRs[95%CI] | *P* | RRs[95%CI] | *P* | RRs[95%CI] | *P* |
| TC, mmol/L | -- | -- | 1.120[1.009,1.230] | 0.033 | -- | -- | -- | -- | 1.130[1.022,1.250] | 0.017 |
| BMI, kg/m^2^ | -- | -- | -- | -- | 0.980[0.945,1.017] | 0.287 | -- | -- | -- | -- |
| PBF, % | -- | -- | -- | -- | -- | -- | 0.979[0.964,0.994] | 0.007 | 0.978[0.963,0.993] | 0.004 |
| Age, year | 0.988[0.979,0.997] | 0.009 | 0.988[0.979,0.997] | 0.007 | 0.987[0.978,0.996] | 0.004 | 0.990[0.981,0.999] | 0.028 | 0.989[0.980,0.998] | 0.022 |
| Hb, g/L | 0.993[0.986,1.000] | 0.088 | 0.992[0.984,0.999] | 0.037 | 0.993[0.986,1.001] | 0.087 | 0.993[0.986,1.000] | 0.084 | 0.991[0.983,0.999] | 0.023 |
| TCO2, mmol/L | -- | -- | -- | -- | -- | -- | 0.992[0.953,1.030] | 0.685 | 0.987[0.949,1.030] | 0.540 |
| Urea, mmol/L | 1.021[0.994,1.050] | 0.124 | 1.020[0.988,1.040] | 0.271 | 1.030[1.000,1.053] | 0.054 | 1.020[0.993,1.050] | 0.144 | 1.010[0.987,1.040] | 0.331 |
| UPCR, mg/g | 1.239[1.185,1.290] | <0.000 | 1.200[1.133,1.260] | 0.000 | 1.240[1.190,1.300] | <0.000 | 1.230[1.172,1.280] | <0.000 | 1.180[1.119,1.250] | 0.000 |
| eGFR, ml/min/1.73 m^2^ | 0.960[0.948,0.972] | 0.000 | 0.957[0.945,0.970] | 0.000 | 0.962[0.950,0.974] | 0.000 | 0.960[0.948,0.972] | 0.000 | 0.957[0.945,0.970] | 0.000 |
| K^+^, mmol/L | 1.235[0.984,1.550] | 0.069 | 1.270[1.009,1.590] | 0.041 | 1.250[0.994,1.565] | 0.057 | 1.190[0.936,1.500] | 0.158 | 1.220[0.959,1.540] | 0.106 |
| Na^+^, mmol/L | 0.956[0.910,1.010] | 0.082 | 0.954[0.907,1.000] | 0.067 | -- | -- | 0.959[0.912,1.010] | 0.109 | 0.957[0.910,1.010] | 0.095 |
| Ca^2+^, mmol/L | -- | -- | 0.836[0.353,1.980] | 0.685 | -- | -- | -- | -- | -- | -- |
| P, mmol/L | 2.774[1.650,4.660] | 0.000 | 2.780[1.635,4.720] | 0.000 | 2.770[1.646,4.645] | 0.000 | 2.550[1.518,4.270] | 0.000 | 2.590[1.544,4.330] | 0.000 |

Note:Hemoglobin, Hb, g/L; Triglyceride, TG; total cholesterol, TC; high-density lipoprotein cholesterol,HDL-C; low-density lipoprotein cholesterol, LDL-C; Total carbon dioxide, TCO2; K+; Na+; Ca2+; P; albumin, ALB; Urine protein-to-creatinine, UPCR, mg/g; Body Mass Index, BMI, kg/m2; triceps skinfold thickness, TSKF; mid-arm circumference, MUAC; Body Fat Mass, BFM; Fat Mass Index, FMI; Percent Body Fat, PBF, %; Visceral Fat Area, VFA; Total Body Water, TBW; Intracellular Water, ICW; Extracellular Water, ECW, kg/L; Fat Free Mass, FFM; Fat Free Mass Index, FFMI.

Table 5 Graphs of Lasso regression

|  | Dataset1 | | |
| --- | --- | --- | --- |
| Model1 | 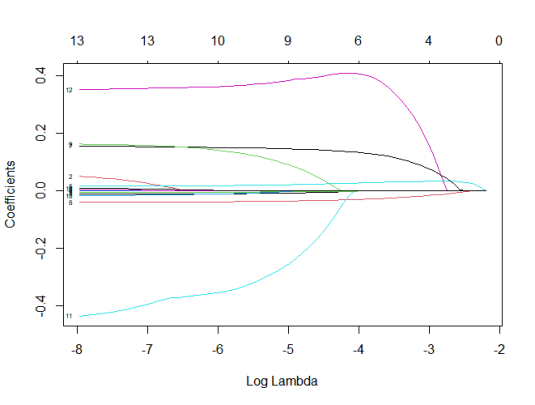 | | 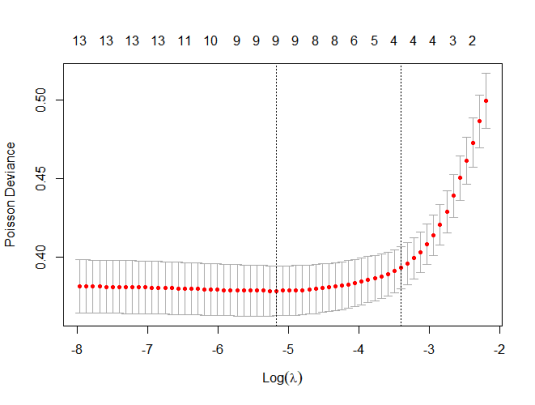 |
| Model2 | 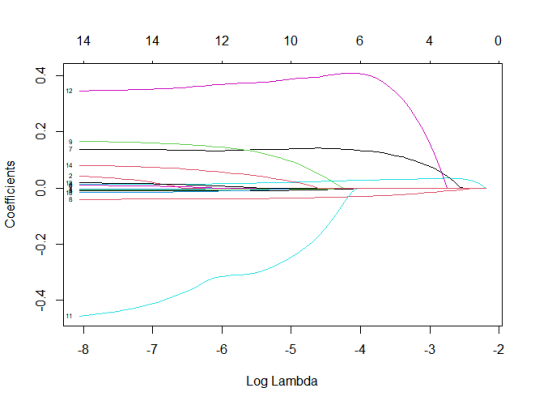 | | 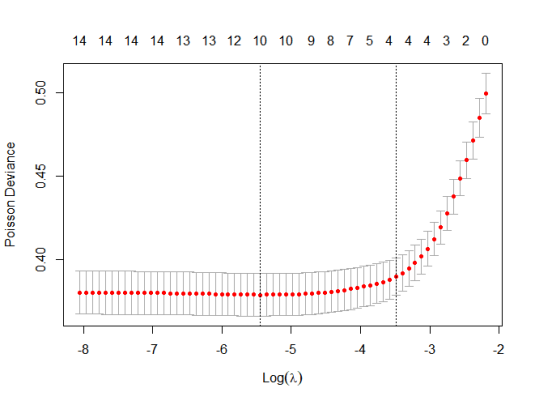 |
| Model3 | 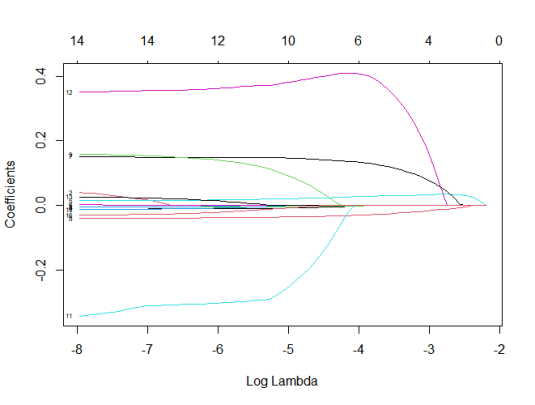 | | 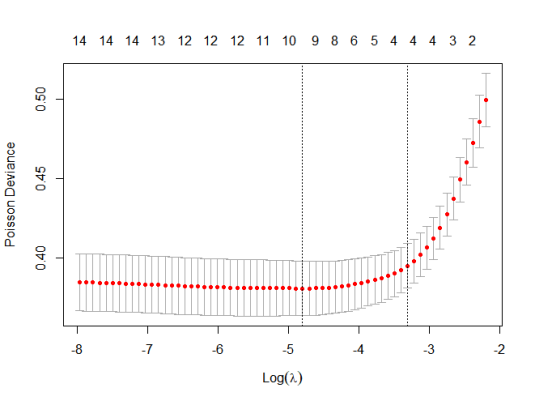 |
| Model4 | 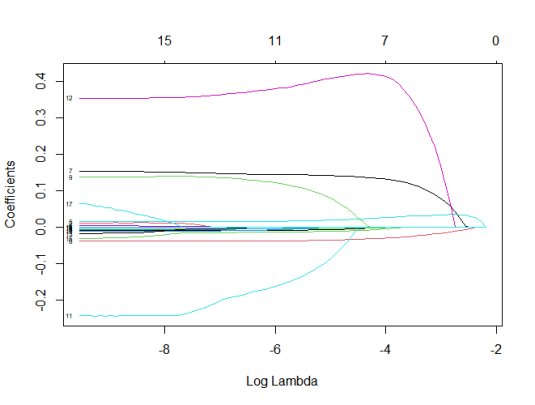 | | 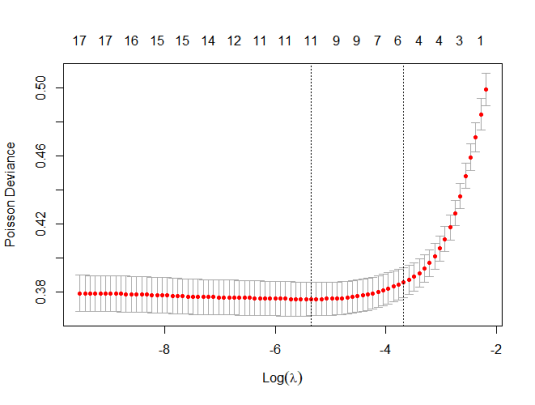 |
| Model5 | 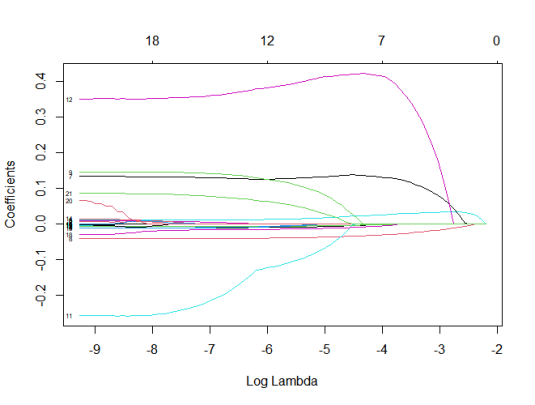 | | 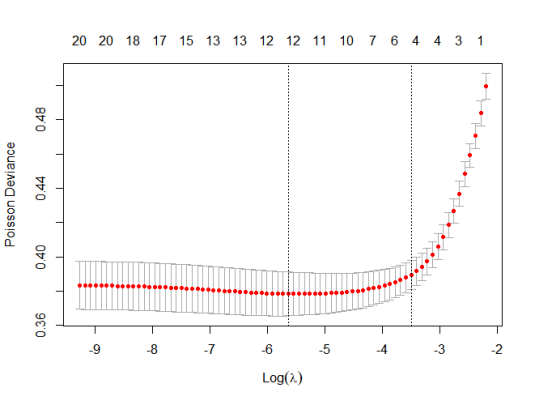 |
|  | Dataset2 | | |
| Model1 | 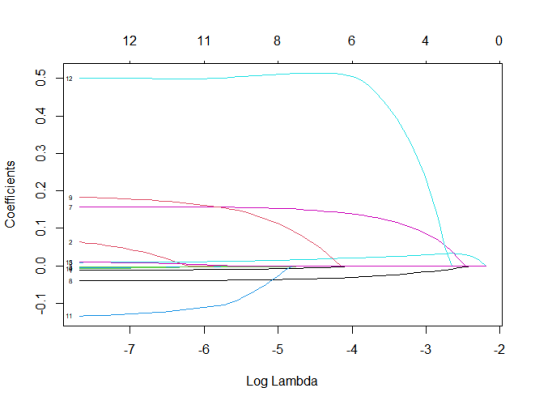 | | 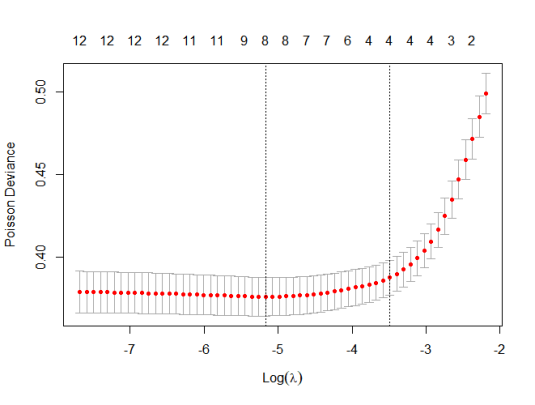 |
| Model2 | 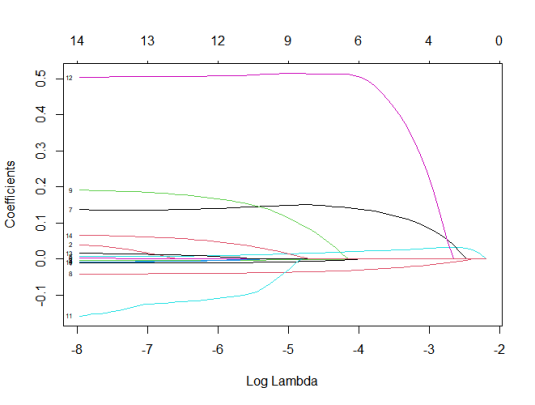 | | 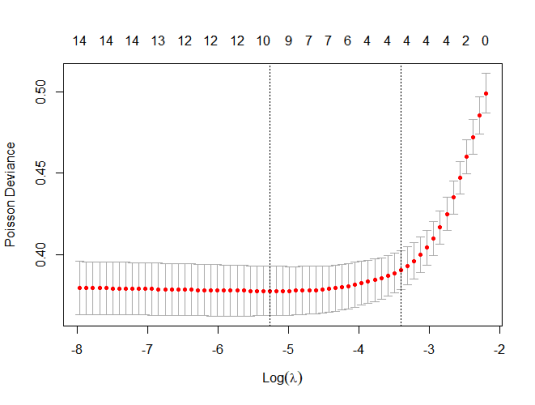 |
| Model3 | 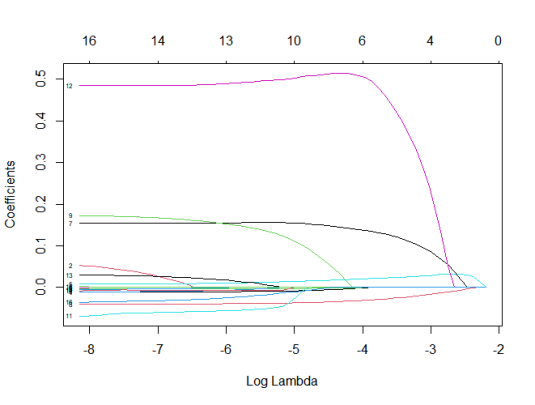 | | 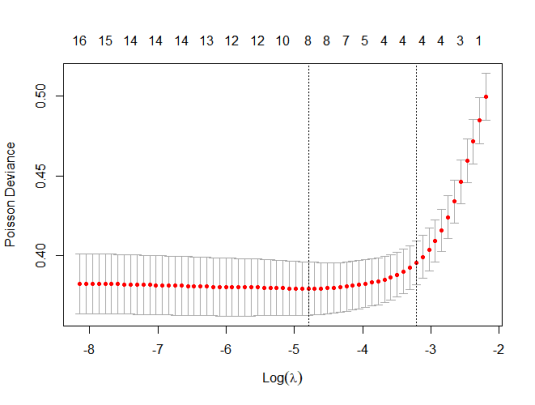 |
| Model4 | 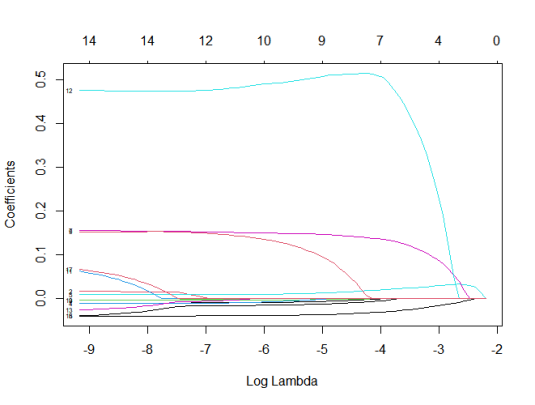 | | 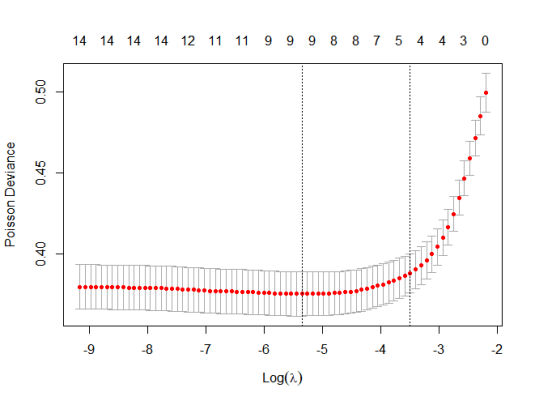 |
| Model5 | 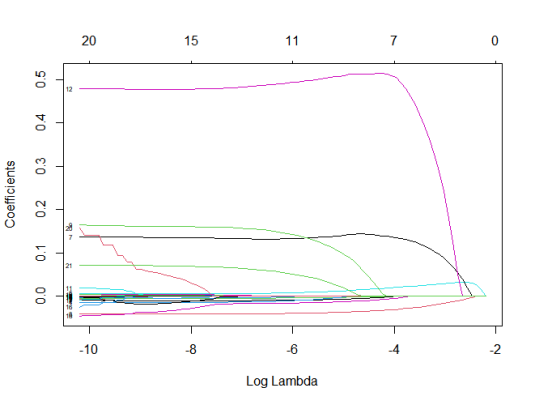 | | 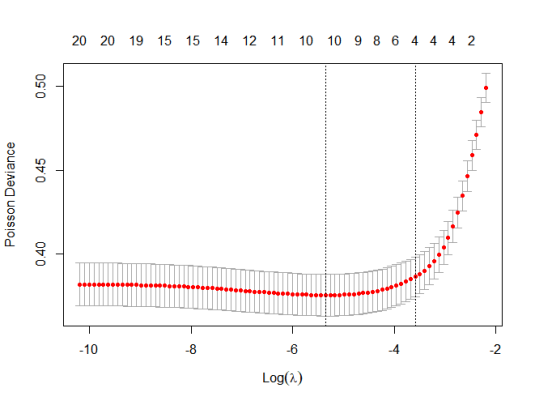 |
|  | Dataset3 | | |
| Model1 | 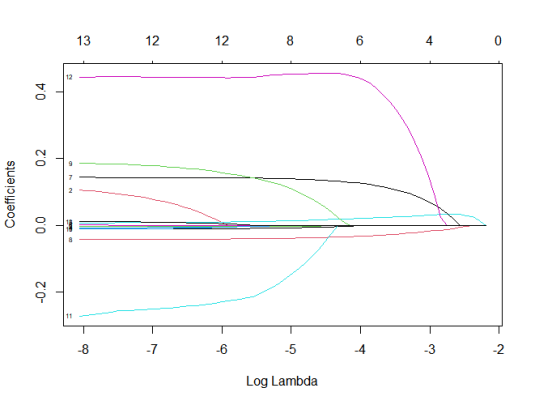 | | 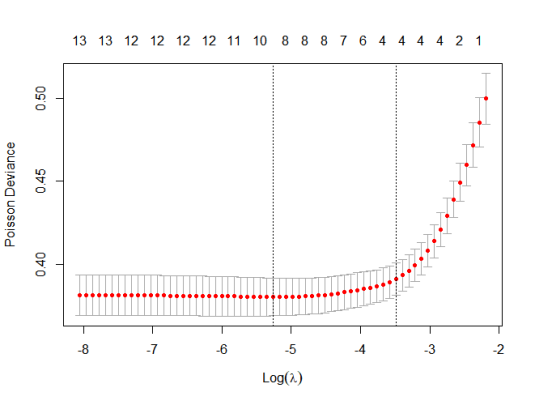 |
| Model2 | 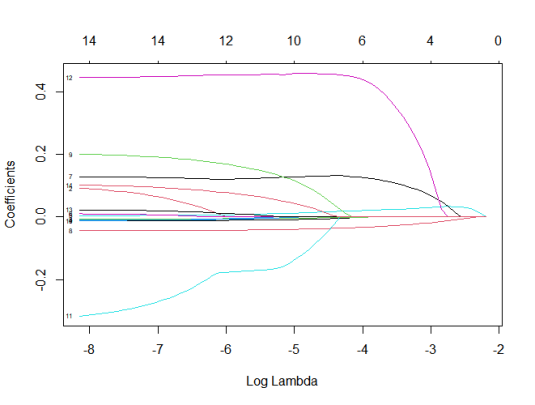 | | 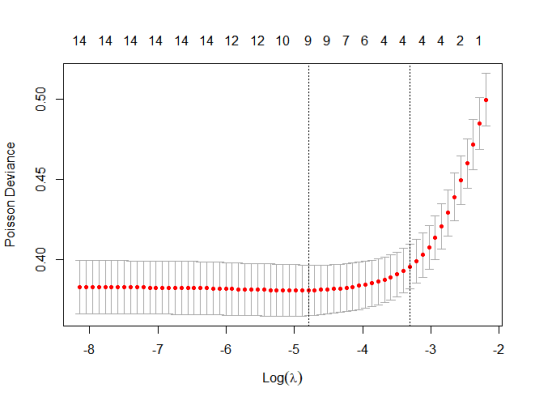 |
| Model3 | 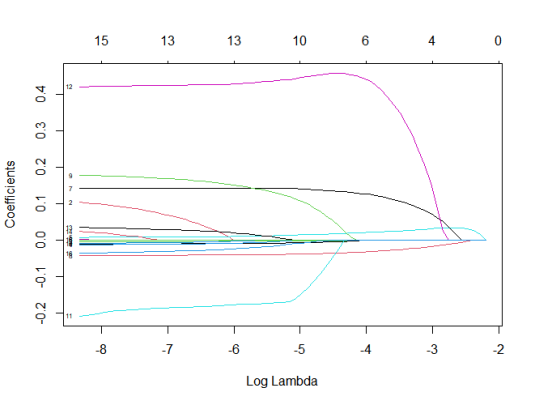 | | 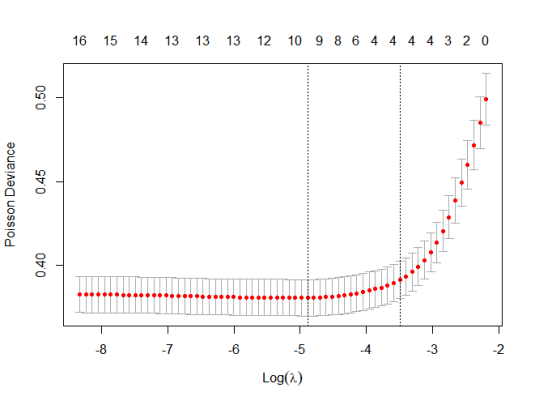 |
| Model4 | 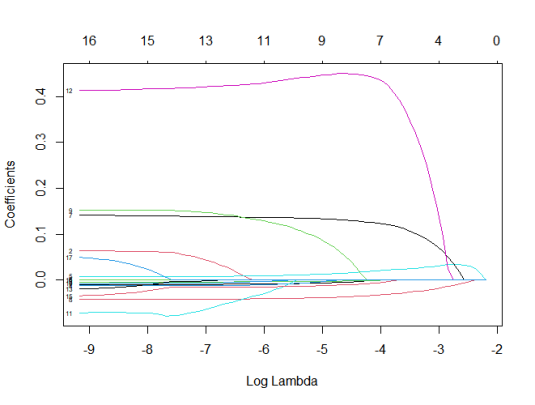 | | 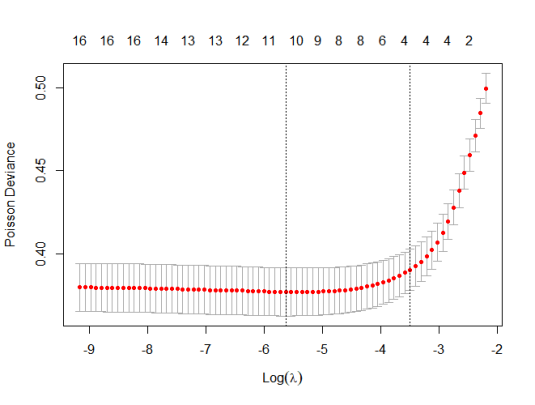 |
| Model5 | 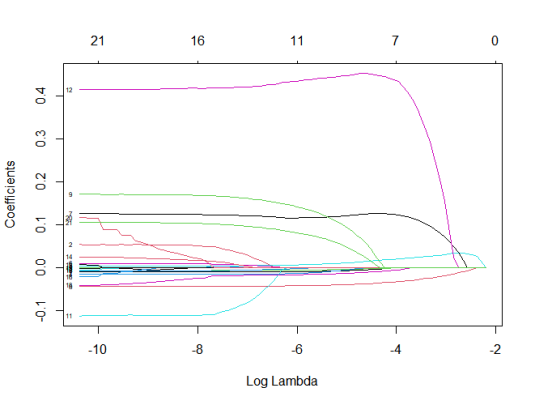 | | 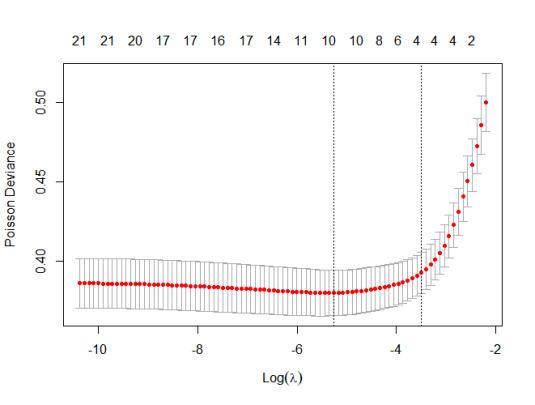 |
|  | Dataset4 | | |
| Model1 | 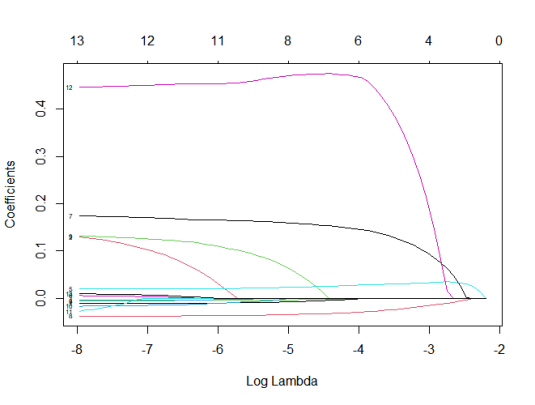 | | 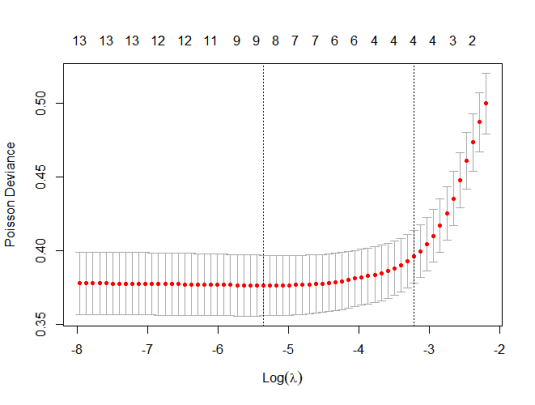 |
| Model2 | 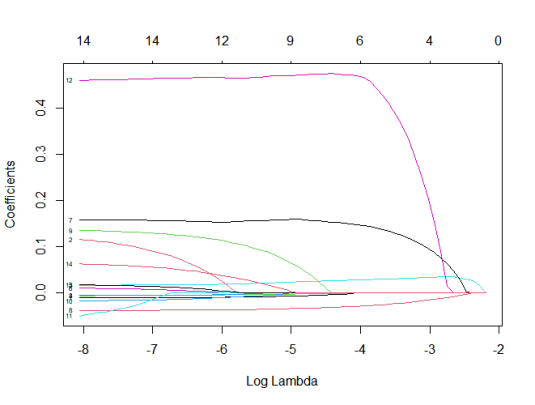 | | 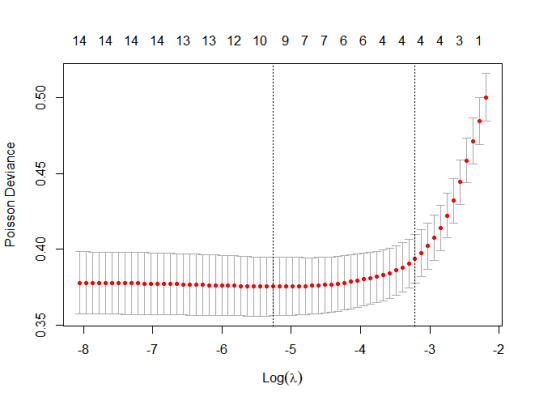 |
| Model3 | 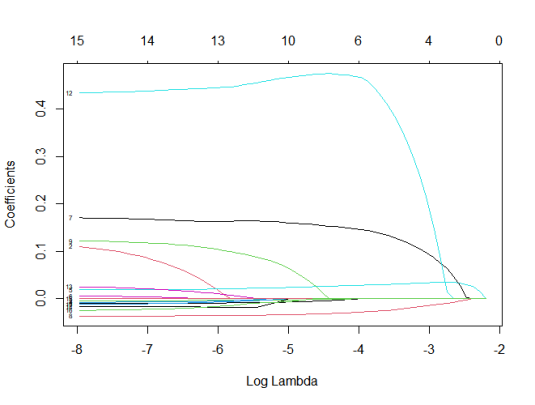 | | 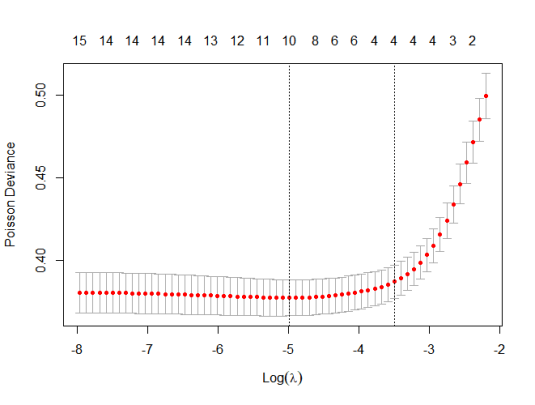 |
| Model4 | 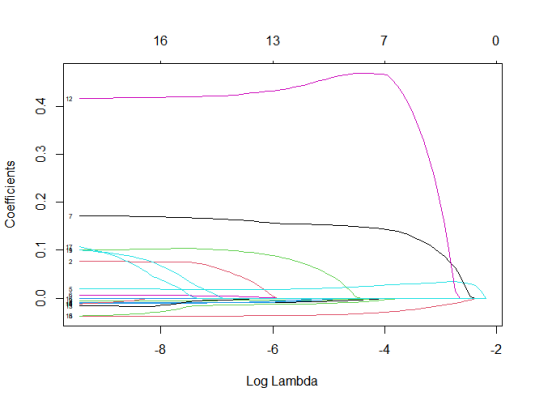 | | 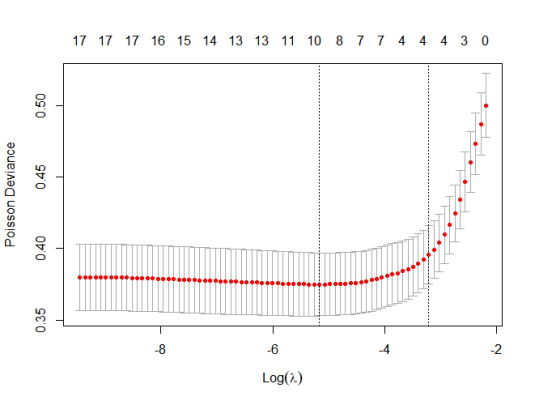 |
| Model5 | 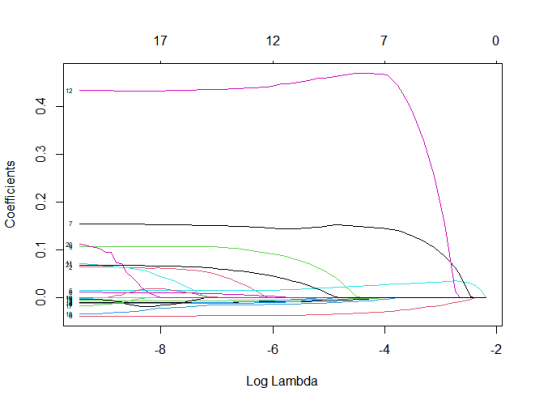 | | 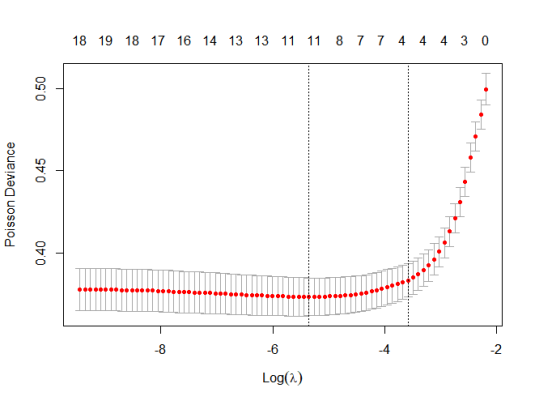 |
|  | Dataset5 | | |
| Model1 | 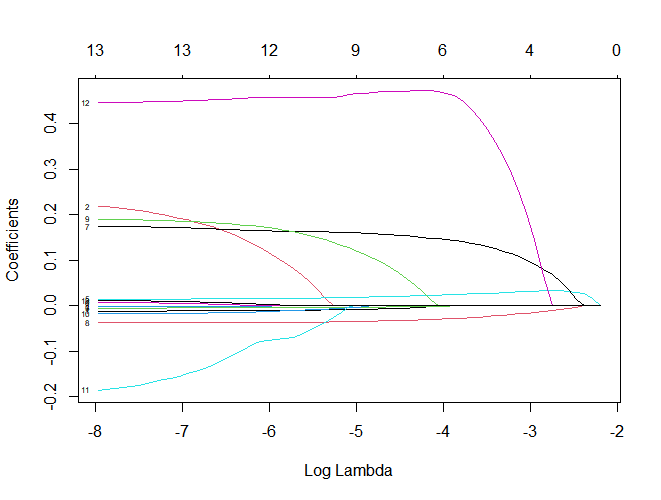 | 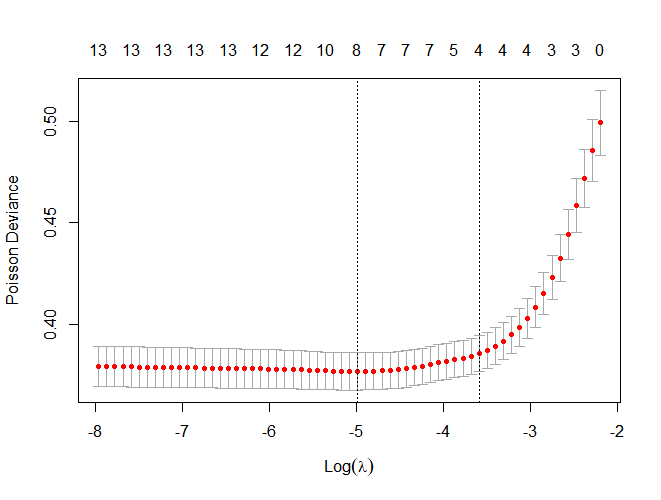 | |
| Model2 | 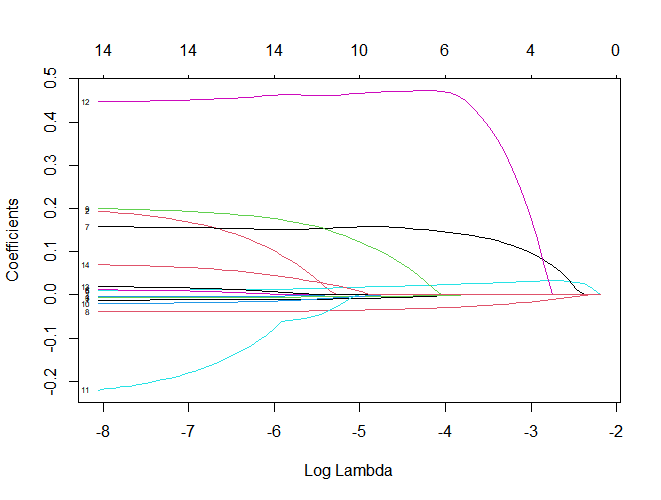 | 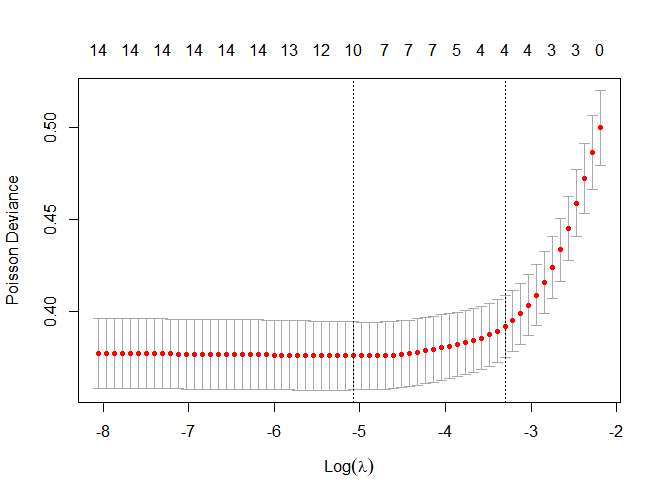 | |
| Model3 | 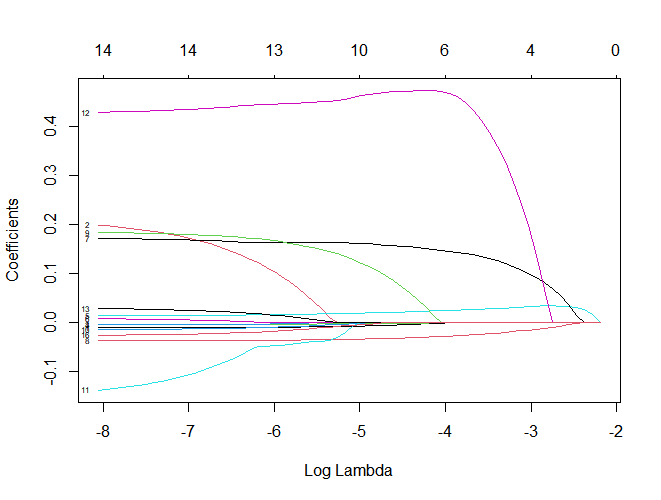 | | 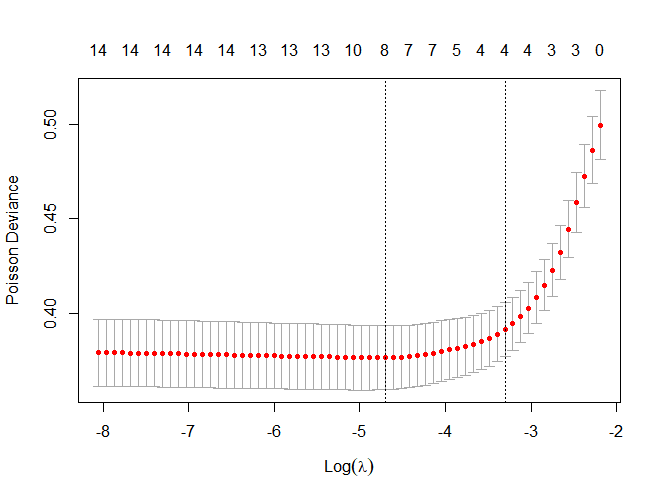 |
| Model4 | 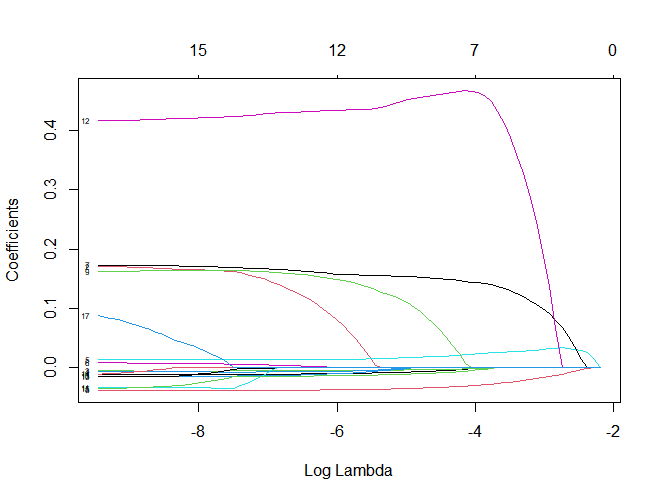 | | 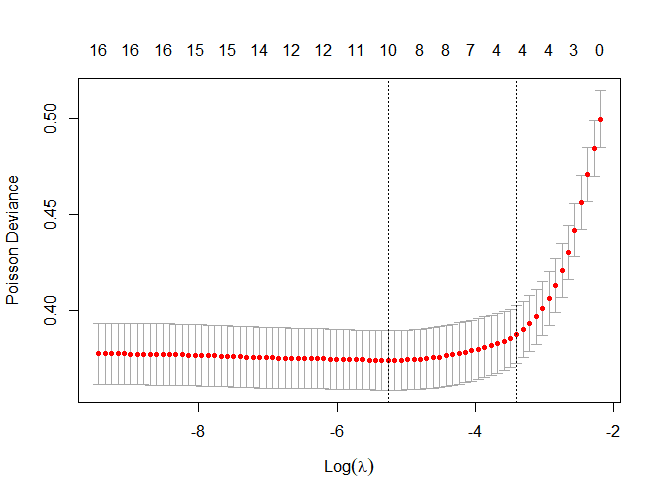 |
| Model5 | 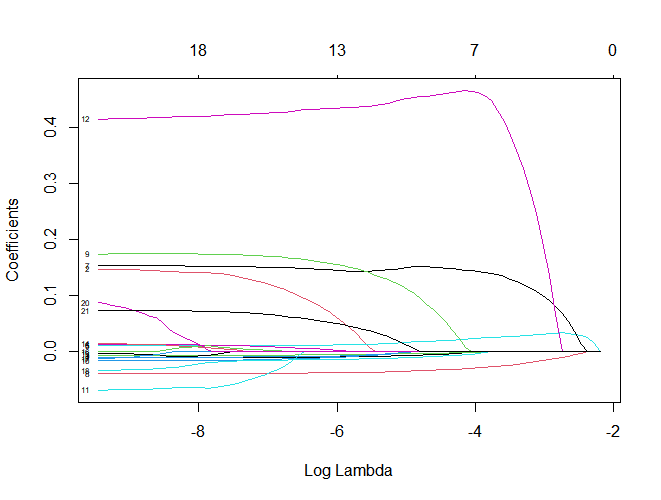 | | 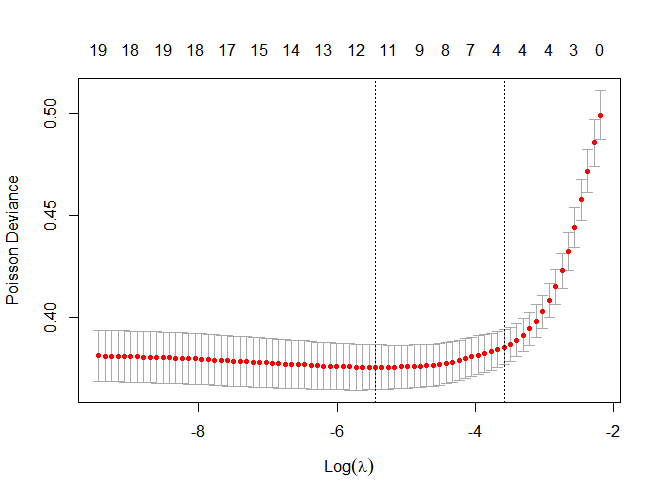 |
