# Supplementary material for: Lipid parameters, adipose tissue distribution and prognosis prediction in chronic kidney Disease patients
Source: Lipids Health Dis. 2024 Jan 8;23:5. doi: 10.1186/s12944-024-02004-4 (PMC10773091; doi:10.1186/s12944-024-02004-4)
Supplement: Supplementary file 7 — Supplementary Material 7 [file 12944_2024_2004_MOESM7_ESM.docx]

**Supplement files 5 Models Comparison**

Table1 Models Comparison of Dataset1

|  | Model 1 | Model 2 | Model 3 | Model 4 | Model 5 |
| --- | --- | --- | --- | --- | --- |
| AIC | 1673.100 | 1669.3 | 1674.20 | 1669.60 | 1664.10 |
| AUC | 0.705[0.671,0.740] | 0.708[0.673,0.742] | 0.705[0.670,0.740] | 0.710[0.676,0.745] | 0.714[0.680,0.748] |
| *P* for ROC | 0.016 | 0.019 | 0.006 | 0.215 | Ref |
| NRI | -0.020[-0.058,0.026] | -0.010[-0.058,0.023] | -0.020[-0.064,0.033] | 0.002[-0.037,0.028] | Ref |


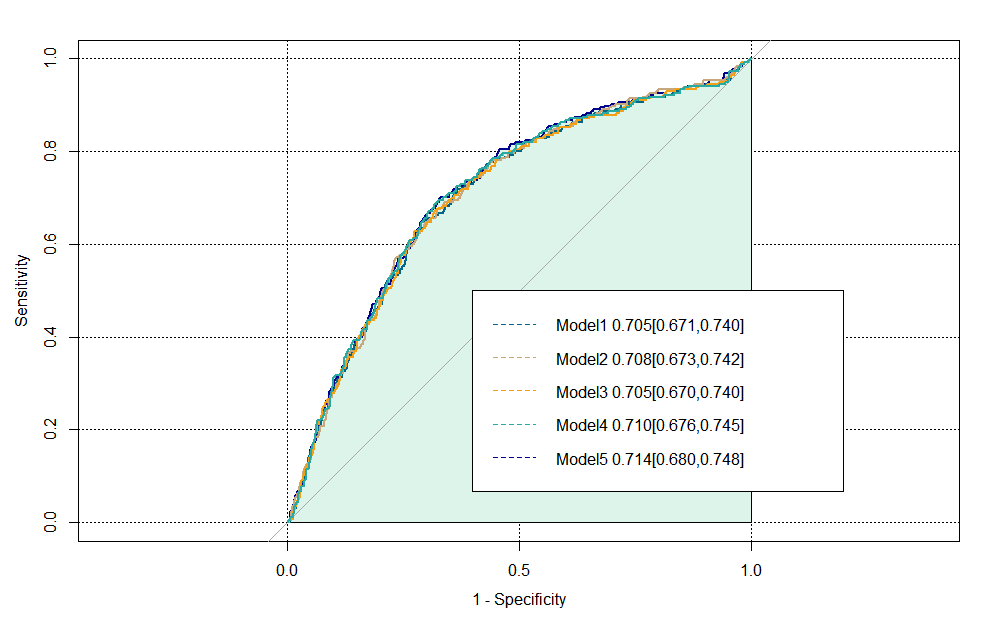


Figure1 AUC of Dataset1

Table2 Models Comparison of Dataset2

|  | Model 1 | Model 2 | Model 3 | Model 4 | Model 5 |
| --- | --- | --- | --- | --- | --- |
| AIC | 1665.70 | 1663.00 | 1663.60 | 1658.30 | 1654.40 |
| AUC | 0.707[0.673,0.742] | 0.709[0.675,0.743] | 0.709[0.674,0.743] | 0.714[0.679,0.748] | 0.716[0.682,0.750] |
| *P* for ROC | 0.010 | 0.011 | 0.010 | 0.386 | Ref |
| NRI | -0.027[-0.060,0.034] | -0.008[-0.037,0.058] | -0.005[-0.040,0.063] | 0.015[-0.035,0.066] | Ref |


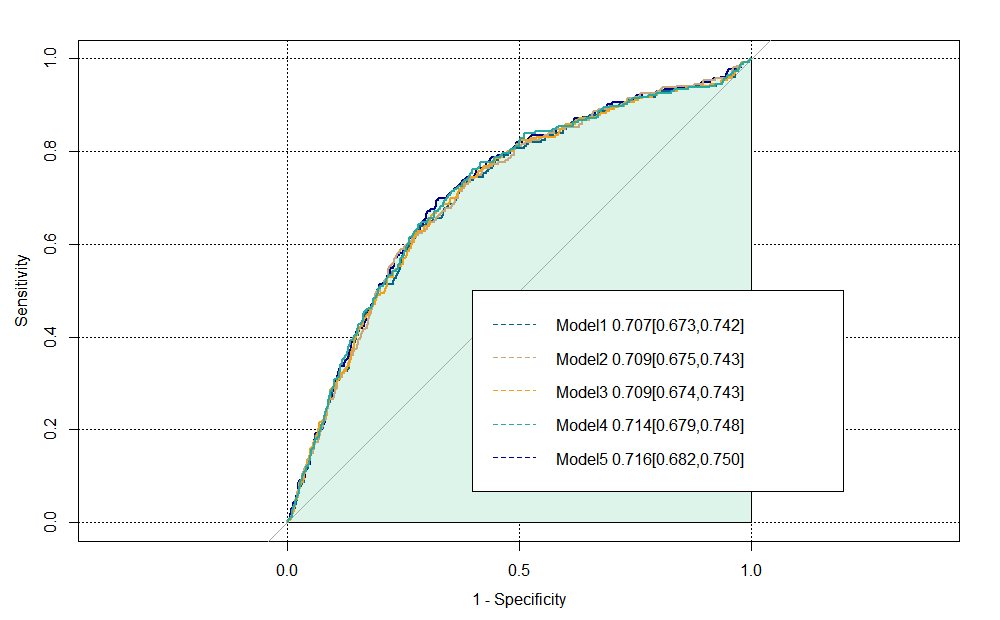


Figure2 AUC of Dataset2

Table3 Models Comparison of Dataset3

|  | Model 1 | Model 2 | Model 3 | Model 4 | Model 5 |
| --- | --- | --- | --- | --- | --- |
| AIC | 1686.20 | 1675.60 | 1684.80 | 1680.70 | 1667.20 |
| AUC | 0.696[0.661,0.731] | 0.702[0.668,0.736] | 0.698[0.663,0.733] | 0.703[0.669,0.738] | 0.709[0.675,0.743] |
| *P* for ROC | 0.003 | 0.014 | 0.002 | 0.132 | Ref |
| NRI | -0.008[-0.061,0.028] | -0.019[-0.058,0.025] | -0.014[-0.055,0.033] | 0.010[-0.049,0.045] | Ref |


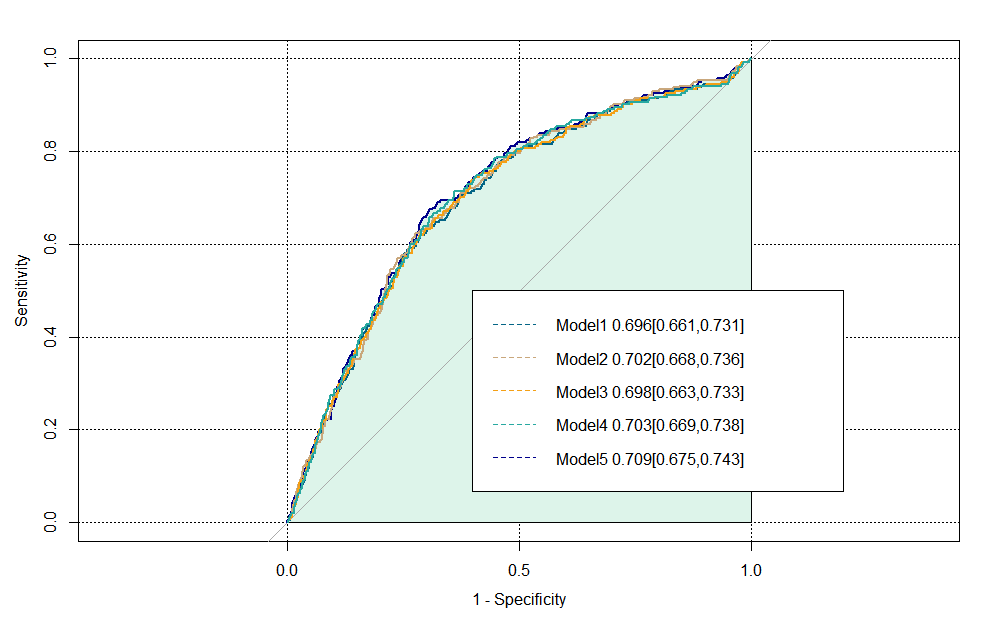


Figure3 AUC of Dataset3

Table4 Models Comparison of Dataset4

|  | Model 1 | Model 2 | Model 3 | Model 4 | Model 5 |
| --- | --- | --- | --- | --- | --- |
| AIC | 1660.80 | 1659.40 | 1660.30 | 1655.80 | 1653.30 |
| AUC | 0.711[0.676,0.745] | 0.712[0.678,0.746] | 0.712[0.678,0.747] | 0.716[0.681,0.750] | 0.718[0.684,0.752] |
| *P* for ROC | 0.018 | 0.018 | 0.048 | 0.263 | Ref |
| NRI | -0.002[-0.050,0.031] | 0.005[-0.048,0.025] | -0.008[-0.048,0.038] | 0.009[-0.035,0.026] | Ref |


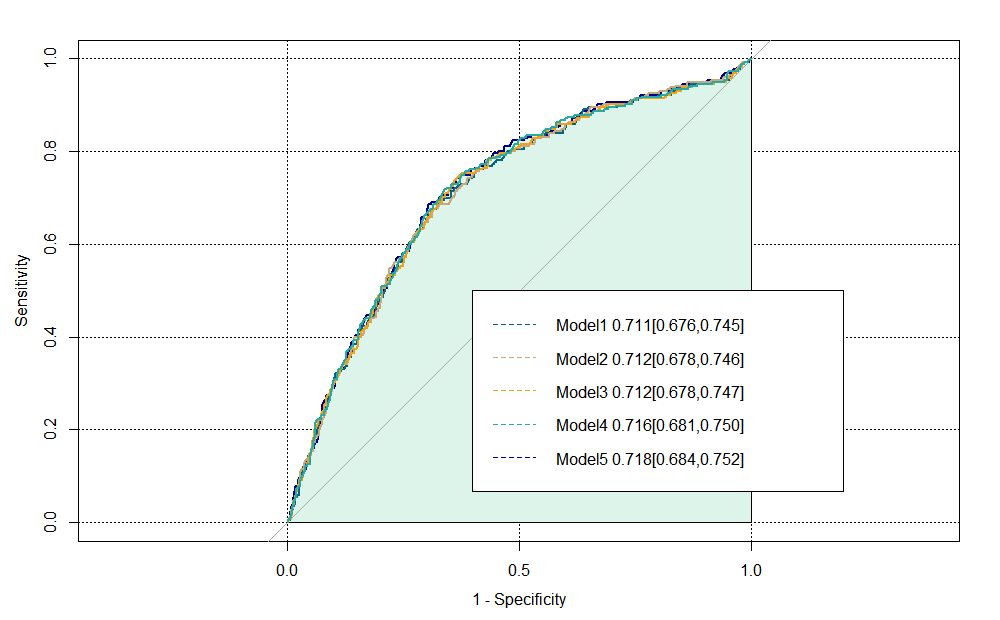


Figure4 AUC of Dataset4

Notes: Akaike information criterion, AIC; the area under the receiver operating curve, AUC; receiver operating curve, ROC; net reclassification index, NRI.

Table 5 Models Comparison of Dataset5

|  | Model 1 | Model 2 | Model 3 | Model 4 | Model 5 |
| --- | --- | --- | --- | --- | --- |
| AIC | 1655.70 | 1655.20 | 1657.50 | 1652.40 | 1649.00 |
| AUC | 0.712[0.678,0.747] | 0.714[0.680,0.748] | 0.712[0.677,0.746] | 0.718[0.684,0.752] | 0.720[0.686,0.754] |
| *P* for ROC | 0.014 | 0.010 | 0.006 | 0.320 | Ref |
| NRI | -0.004[-0.041,0.055] | -0.003[-0.041,0.056] | 0.006[-0.040,0.055] | 0.014[-0.032,0.064] | Ref |


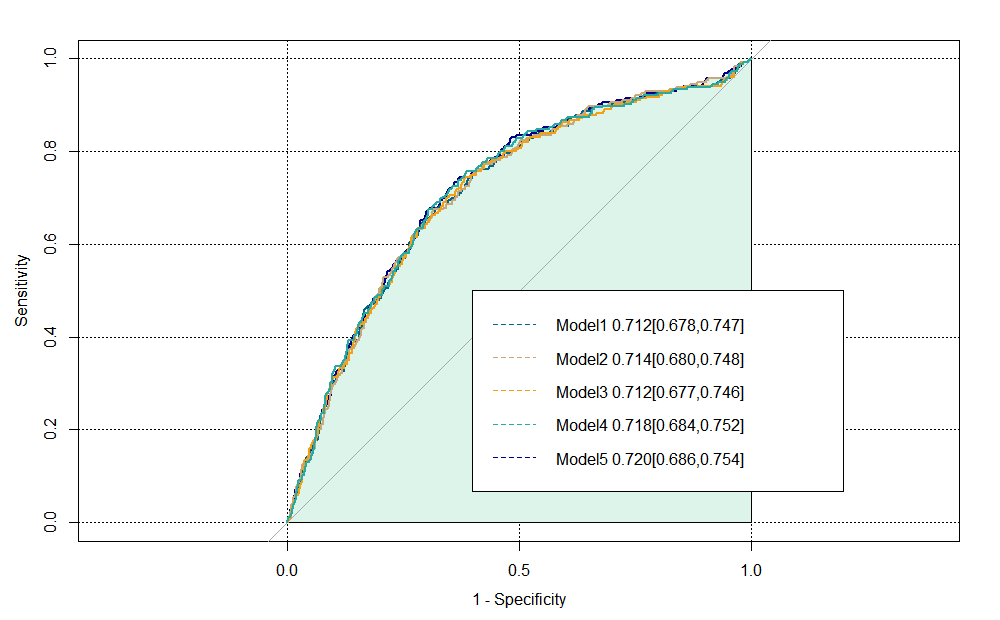


Figure5 AUC of Dataset5
